# The histone H2B ubiquitin ligase RNF40 is required for HER2-driven mammary tumorigenesis

**DOI:** 10.1038/s41419-020-03081-w

**Published:** 2020-10-17

**Authors:** Florian Wegwitz, Evangelos Prokakis, Anastasija Pejkovska, Robyn Laura Kosinsky, Markus Glatzel, Klaus Pantel, Harriet Wikman, Steven A. Johnsen

**Affiliations:** 1 Department of General, Visceral and Pediatric Surgery, University Medical Center Göttingen, Göttingen, Germany; 2 Department of Gynecology and Obstetrics, University Medical Center Göttingen, Göttingen, Germany; 3 Institute for Neuropathology, University of Hamburg-Eppendorf, Hamburg, Germany; 4 Institute of Tumor Biology, University Medical Center Hamburg-Eppendorf, Hamburg, Germany; 5 Gene Regulatory Mechanisms and Molecular Epigenetics Lab, Division of Gastroenterology and Hepatology, Mayo Clinic, Rochester, MN USA

**Keywords:** Breast cancer, Histone post-translational modifications, Transcriptional regulatory elements

## Abstract

The HER2-positive breast cancer subtype (HER2^+^-BC) displays a particularly aggressive behavior. Anti-HER2 therapies have significantly improved the survival of patients with HER2^+^-BC. However, a large number of patients become refractory to current targeted therapies, necessitating the development of new treatment strategies. Epigenetic regulators are commonly misregulated in cancer and represent attractive molecular therapeutic targets. Monoubiquitination of histone 2B (H2Bub1) by the heterodimeric ubiquitin ligase complex RNF20/RNF40 has been described to have tumor suppressor functions and loss of H2Bub1 has been associated with cancer progression. In this study, we utilized human tumor samples, cell culture models, and a mammary carcinoma mouse model with tissue-specific *Rnf40* deletion and identified an unexpected tumor-supportive role of RNF40 in HER2^+^-BC. We demonstrate that RNF40-driven H2B monoubiquitination is essential for transcriptional activation of RHO/ROCK/LIMK pathway components and proper actin-cytoskeleton dynamics through a trans-histone crosstalk with histone 3 lysine 4 trimethylation (H3K4me3). Collectively, this work demonstrates a previously unknown essential role of RNF40 in HER2^+^-BC, revealing the H2B monoubiquitination axis as a possible tumor context-dependent therapeutic target in breast cancer.

## Introduction

Breast cancer (BC) is the most common form of cancer in the female population^1^. The survival rates of BC vary greatly and strongly depend on both early detection as well as the molecular subtype^2^. Notably, the HER2-positive breast cancer subtype (HER2^+^-BC) is particularly invasive and displays a poorer prognosis compared to hormone receptor-positive (ER+ or/and PR+) BC^3^. Importantly, while current anti-HER2 therapies are initially highly effective for many BC patients with HER2^+^-tumors, a significant number of patient tumors develop therapy resistance, tumor relapse, and disease progression^4^. Thus, new approaches are necessary to combat HER2^+^-BC.

Precision oncology approaches aim to utilize or develop novel targeted therapies that exploit tumor-specific dependencies and/or vulnerabilities based on specific molecular alterations present in a given tumor or molecular subtype^5^. Similar to genetic alterations, epigenetic alterations play an important role in tumorigenesis and tumor progression, resulting in altered patterns of DNA methylation, post-translational histone modifications, and changes in chromatin accessibility or chromatin architecture. Due to the reversible nature of many of these changes, numerous substances targeting epigenetic factors are currently in various stages of preclinical and clinical testing to determine their efficacy as anticancer therapies^6^.

Previous work from our lab and others revealed a particular importance of histone 2B monoubiquitination (H2Bub1) in controlling cellular differentiation^7–10^ and demonstrated that H2Bub1 levels are decreased in ER-positive BC compared to normal adjacent epithelium^11,12^. These findings have led to the hypothesis that H2Bub1, catalyzed by the obligate heterodimeric Ring Finger Protein 20 and 40 (RNF20/RNF40) E3 ubiquitin ligase complex, has a tumor-suppressive function. This hypothesis has been further supported by studies investigating the function of RNF20 and RNF40^13–17^, while we and others have uncovered tumor-supportive roles of RNF20 and RNF40 in colorectal cancer^18,19^ and androgen-dependent prostate cancer^20^, suggesting that RNF20/RNF40-driven H2B monoubiquitination plays a context-dependent role in cancer.

H2Bub1 is localized across the body of active genes^21^ and is closely coupled to transcriptional elongation^22–25^. Studies in both yeast and human cells have revealed a coupling of H2Bub1 and the trimethylation of lysines 4 and 79 of histone 3 (H3K4me3 and H3K79me3, respectively) near the transcriptional start site and transcribed regions of active genes, respectively^22,26–30^. Interestingly, past studies demonstrated that H3K4me3 extends into the transcribed region of genes displaying a particularly high transcriptional elongation rate^31^. Consistent with H2Bub1 being closely linked to transcriptional elongation^23^, we recently demonstrated that loss of RNF40-mediated H2B monoubiquitination results in the narrowing of H3K4me3 domains near the transcriptional start sites (TSS) of important cell fate-determining genes displaying high elongation rates^10,22,32^.

In this study, we sought to examine the role of RNF40-mediated H2B monoubiquitination in HER2^+^-BC. Our studies using a tissue-specific transgenic and gene ablation approach demonstrate for the first time that RNF40 exerts a profound tumor-supportive function in HER2-driven mammary carcinoma. In support of these in vivo findings, we show that RNF40 silencing leads to decreased cell proliferation and specific transcriptional and epigenetic changes in human HER2^+^-BC cell lines. Finally, we unveil a previously undescribed role of RNF40-mediated H2B monoubiquitination in driving the expression of specific genes regulating actin-cytoskeleton dynamics and downstream signaling.

## Materials and methods

### Animal handling and mouse model generation

Animals were housed under specific pathogen-free (SPF) conditions in accordance with the animal rights laws and regulations of Lower-Saxony (LAVES, registration number #15/1754). See also Supplementary Data for more details.

### Histology of human and murine tissues and publically available dataset analyses

Tissue microarrays of human primary and metastatic breast cancer were generated at the University Medical Center Hamburg Eppendorf, Germany (local ethical committee approval number: OB/V/03 and MC-267/13, respectively) in accordance with the ethical standards of the 1964 Declaration of Helsinki. RNF40 and H2Bub1 scoring were established based on the staining intensity (null = no detectable staining, low = weak staining intensity, high = strong staining intensity). Detailed staining procedures and antibodies used for immunohistochemical staining are provided in Supplementary Information.

### Publically available datasets

The Kaplan–Meier plotter (kmplot.com) and The Cancer Genome Atlas (https://portal.gdc.cancer.gov/)-derived publically available datasets were used to examine the association of *RNF40* expression with Relapse-Free Survival (RFS) or Overall Survival (OS) of HER2^+^-BC patients. Parameters for BC subtype classification are given in Supplementary Information. Publically available ChIP-seq datasets for HCC1954 cells (GSE85158 and GSE72956) were downloaded from Gene Expression Omnibus (www.ncbi.nlm.nih.gov/geo/)^33,34^.

### Cell culture, transfections, and functional assays

HCC1954 (CRL-2338™) and SKBR3 (HTB-30™) cells were purchased from the American Type Culture Collection (ATCC®). siRNA transfections were performed using Lipofectamine^®^ RNAiMAX (Invitrogen) according to the manufacturer’s guidelines. Proliferation kinetics and tumorspheres were recorded using Celigo^®^ S imaging cytometer (Nexcelom Bioscience LLC) and IncuCyte^®^ Live Cell Analysis System (Sartorius AG). Colonies and migrated cells from transwell assay were washed with PBS, fixed, stained and scanned with an Epson Perfection V700 Photo. Further details are available in Supplementary Data.

### Immunofluorescence microscopy

Cells were plated and transfected on coverslips and grown for another 72 h. Cells were then washed with PBS, cross-linked with 4% paraformaldehyde and permeabilized with 1% Triton X-100 in PBS or TBS for 10 min, blocked for 1 h and incubated with the primary antibody overnight. Coverslips were washed and secondary antibody was applied with DAPI for 1 h at room temperature. Coverslips were washed and mounted on microscope slides. A detailed protocol and a list of antibodies are available in Supplementary Data.

### Microscopy

Immunohistochemistry (IHC) pictures were taken with a Zeiss Axio Scope A1. Bright-field images of cultured cells were taken with a Nikon Eclipse S100 inverted microscope and immunofluorescence pictures with a Zeiss LSM 510 Meta confocal microscope. Fluorescence intensity was quantified using the ImageJ software. Image analysis workflow is described in the Supplementary Data.

### Annexin and caspase 3/7 activity assay

For annexin V staining, cells were trypsinized and resuspended in binding buffer at 72 h post-transfection and incubated with Annexin-V-FITC (Southern Biotech) and propidium iodide (Sigma–Aldrich) for 15 min at room temperature. Samples were analyzed using a Guava EasyCyte Plus flow cytometer (Guava Technologies).

The kinetic apoptosis assay for caspase 3/7 activity was performed according to the manufacturer’s instructions (CS1-V0002(3)-1, ViaStainTM Live Caspase 3/7 Detection Kit, Nexcelom). Scanning was performed 24, 48, and 72 h post-transfection using a Celigo^®^ S imaging cytometer (Nexcelom Bioscience LLC). For detailed protocols, please refer to the Supplementary Data.

### ChIP library preparation and data analysis

Chromatin immunoprecipitation was performed as described previously^35^ 72 h after transfection with control or RNF40 siRNAs using antibodies against H2Bub1 (Cat. No. 5546 S, Cell Signaling Technology) and H3K4me3 (Cat. No. C15410003-50, Diagenode). Next-generation sequencing libraries were prepared using the Microplex Library Preparation kit v2 (Diagenode, Cat.No. C05010011) according to manufacturer’s instructions and samples were sequenced (single-end 50 bp) on a HiSeq4000 (Illumina) at the NGS Integrative Genomics Core Unit (NIG) at the University Medical Center Göttingen (ArrayExpress accession E-MTAB-9234). Processing of sequencing data was performed in the Galaxy environment (galaxy.gwdg.de). Briefly, ChIP-seq reads were mapped to the hg19 reference genome assembly using Bowtie2 (version 2.3.2.2). PCR duplicates were removed using the RmDup tool (version 2.0.1). The deeptools suite (version 3.2.0.0.1) was utilized to generate normalized coverage files (bamCoverage), call peak changes (bigwigCompare), and to generate aggregate plots and heatmaps (computeMatrix and plotHeatmap). Occupancy profiles were visualized using the Integrative Genomics Viewer (IGV 2.4.8). A detailed analysis workflow is available in Supplementary Data.

### RNA library preparation and data analysis

RNA sequencing libraries were generated from HCC1954 cells at 72 h post-transfection with the NEXTFLEX^®^ Rapid Directional RNA-Seq Kit (Bioo Scientific, Catalog #NOVA-5138-07) according to the manufacturer’s instructions and samples were sequenced (single-end 50 bp) on a HiSeq4000 (Illumina) at the NIG (ArrayExpress accession E-MTAB-9234). RNA-seq data were processed in the Galaxy environment. Raw reads were trimmed (FASTQ Trimmer), mapped to the reference genome hg19 using TopHat (version 2.1.1) and read counts per gene were calculated with featureCounts. Finally, differential gene expression analysis and normalized counts were obtained using DESeq2. A detailed analysis workflow is available in Supplementary Data.

## Results

### RNF40 is highly expressed in HER2^+^-BC

**Fig. 1:**
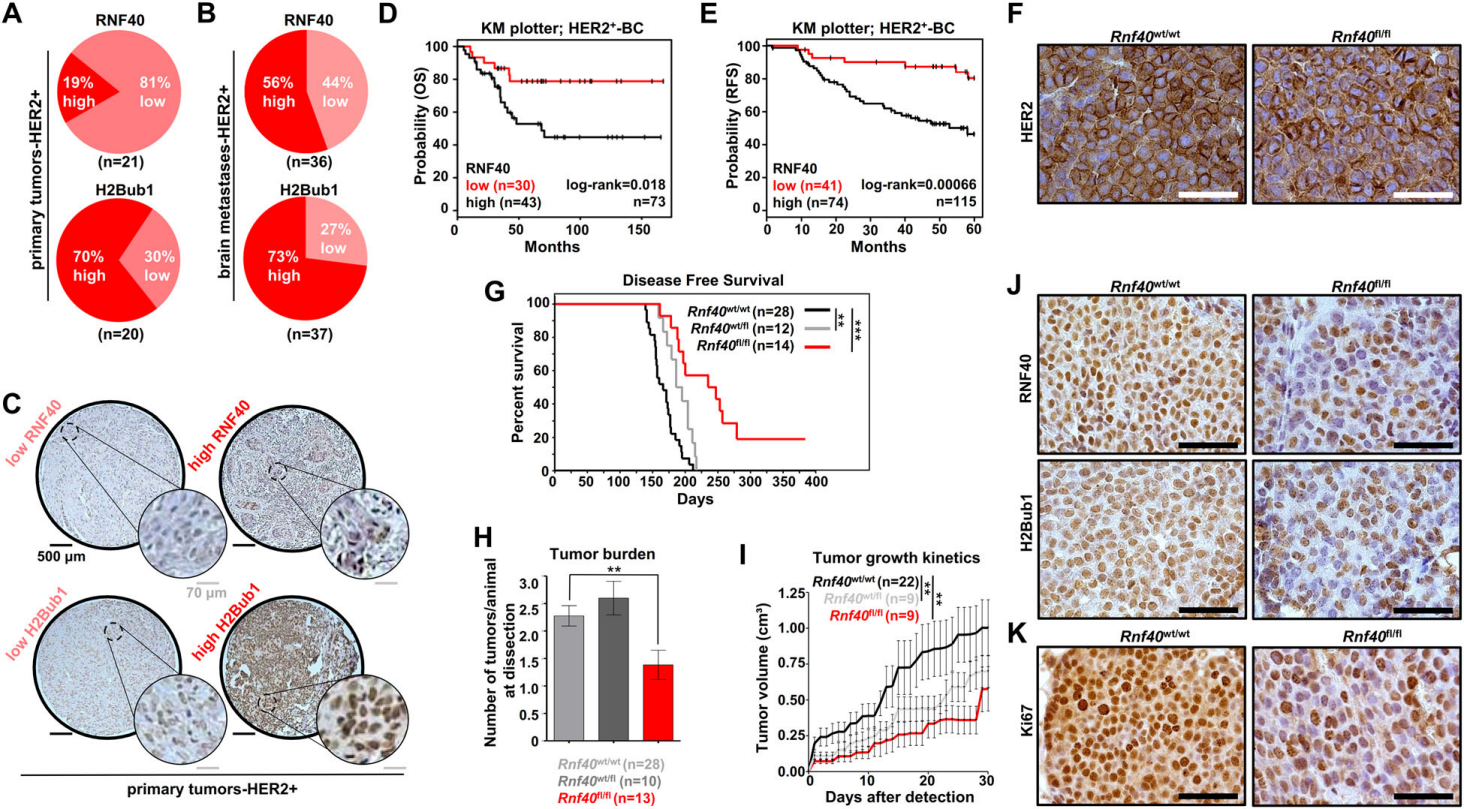
RNF40 and H2Bub1 are maintained in HER2^+^-BC. **A, B** TMAs with HER2-positive primary BC (*n* = 21) and brain metastases (*n* = 37) were stained for RNF40 and H2Bub1 by IHC. Distribution of RNF40 and H2Bub1 staining intensity in HER2-positive primary BCs (**A**) and brain metastases (**B**). **C** Representative images of low and high H2Bub1 and RNF40 staining intensity in primary HER2-BC specimens. **D, E** Overall survival (OS) (**D**) and relapse-free survival (RFS) plots (**E**) of HER2-BC patients with low and high gene expression of *RNF40*, using the online tool kmplot.com. Log-rank test. **F** Representative immunohistochemical staining of HER2 in the *Rnf40*^wt/wt^ and *Rnf40*^fl/fl^ mammary carcinomas. **G** Disease-free survival of *Rnf40*^wt/wt^ compared to *Rnf40*^wt/fl^ or *Rnf40*^fl/fl^ mice. Log-rank test. **H** Bar graph depicting the average number of observed tumors per animal in each transgenic mouse cohort. **I** Tumor growth kinetics of all transgenic mouse cohorts. **J, K** Immunohistochemical detection of RNF40, H2Bub1 (**J**) and Ki67 (**K**) in the *Rnf40*^wt/wt^ and *Rnf40*^fl/fl^ mammary carcinomas. Scale bars: 100 μm. **H**, **I** One-way ANOVA test. **p < 0.01, ***p < 0.005. Error bars: standard error of the mean (SEM).

While we and others have uncovered potential opposing tumor-supportive or tumor-suppressive roles of H2Bub1 and its E3 ligases RNF20 and RNF40 in ER-positive and triple-negative BC^11,12,16^, the role of this epigenetic pathway in HER2^+^-BC is currently unclear. Therefore, we investigated RNF40 expression and H2Bub1 levels by immunohistochemical staining of tissue microarrays containing 21 primary HER2^+^-BC samples and 38 brain metastases. Interestingly, all analyzed HER2^+^-BC samples expressed detectable levels of both RNF40 and H2Bub1 (Fig. 1A–C and Fig. S1A). Notably, high levels of RNF40 expression were more frequently detected in HER2^+^ brain metastases compared to primary tumors (Fig. 1A, B, Fig. S1B). We, therefore, examined the relationship between *RNF40* mRNA levels and survival in HER2^+^-BC patients using publically available data (TCGA, KM plotter) and observed that high levels of *RNF40* expression were associated with reduced overall, relapse-free and distant metastasis-free survival (Fig. 1D, E and Fig. S1C). Unfortunately, due to the limited number of primary HER2^+^-BC samples analyzed in this study, we were not able to detect any correlation of H2Bub1 or RNF40 levels to tumor grade (Fig. S1D). Interestingly, *RNF40* expression was found to be significantly higher in HER2^+^-BC tissues compared to normal mammary tissues in the TCGA dataset (Fig. S1E). In summary, these data demonstrate that RNF40 expression is not lost in metastatic HER2^+^-BC and that its expression correlates with poor prognosis in these patients.

### RNF40 plays a tumor-supportive function in *Erbb2*-driven mammary carcinoma in vivo

Since RNF40 expression and activity were largely maintained in human HER2^+^-BC, we hypothesized that a loss of RNF40 may impair HER2-driven tumorigenesis. To test this hypothesis we utilized the MMTV-*Erbb2* genetic mouse model^36^ to generate a tri-transgenic MMTV-*Erbb2;* MMTV-Cre; *Rnf40*^flox^ mouse line with mammary tissue-specific co-expression of HER2 and Cre recombinase, and a floxed *Rnf40* allele. This approach enabled us to achieve a simultaneous HER2 overexpression and mammary epithelium-specific ablation of *Rnf40*^18,22^. Consistent with our findings in human HER2^+^-BC, MMTV-*Erbb2; Rnf40*^wt/wt^ tumors did not display a loss of either RNF40 or H2Bub1 when compared to the adjacent normal mammary epithelium (Fig. S1F). Moreover, immunohistochemical analyses confirmed that HER2 expression was unaffected by *Rnf40* deletion (Fig. 1F). However, both heterozygous *(Rnf40*^wt/fl^), and especially homozygous loss of *Rnf40 (Rnf40*^fl/fl^) resulted in a pronounced increase of tumor-free survival of MMTV-*Erbb2* animals (Fig. 1G). Remarkably, despite the high tumor incidence in this mouse model (100% of *Rnf40*^wt/wt^ mice developed tumors within 220 days), 2 out of 14 *Rnf40*^fl/fl^ animals (14%) never developed tumors within 18 months (Fig. 1G). Moreover, *Rnf40*^fl/fl^ mice developed significantly fewer tumors than *Rnf40*^wt/wt^ (Fig. 1H) and displayed strongly reduced tumor growth kinetics (Fig. 1I). Notably, *Rnf40* loss did not induce morphological changes, as visible in H&E staining of the *Rnf40*^wt/wt^ and *Rnf40*^fl/fl^ tumors (Fig. S1H). To estimate the efficiency of *Rnf40* deletion in this model, we performed qRT-PCRs and immunohistochemical staining in *Rnf40*^wt/wt^ and *Rnf40*^fl/fl^ tumors (Fig. 1J and S1G). Consistent with the lack of a complete block in tumor incidence and growth, *Rnf40*^fl/fl^ lesions displayed a heterogeneous pattern of RNF40 expression, suggesting that the few tumors that did develop in this model were largely caused by an incomplete deletion of the *Rnf40* allele. Consistently, similar effects have been reported in several other tumor types and with various Cre models, where rare tumors that appeared consistently retained some expression of the essential floxed gene^37,38^. This is further supported by the observation that both H2Bub1, as well as the proliferation marker Ki67, displayed a similar heterogeneous expression pattern as RNF40 (Fig. 1J, K and Fig. S1I). Taken together, these results demonstrate that RNF40 plays an essential role in HER2-driven mammary tumor initiation and progression.

### RNF40 loss impairs oncogenic properties of HER2^+^-BC cells in vitro

**Fig. 2:**
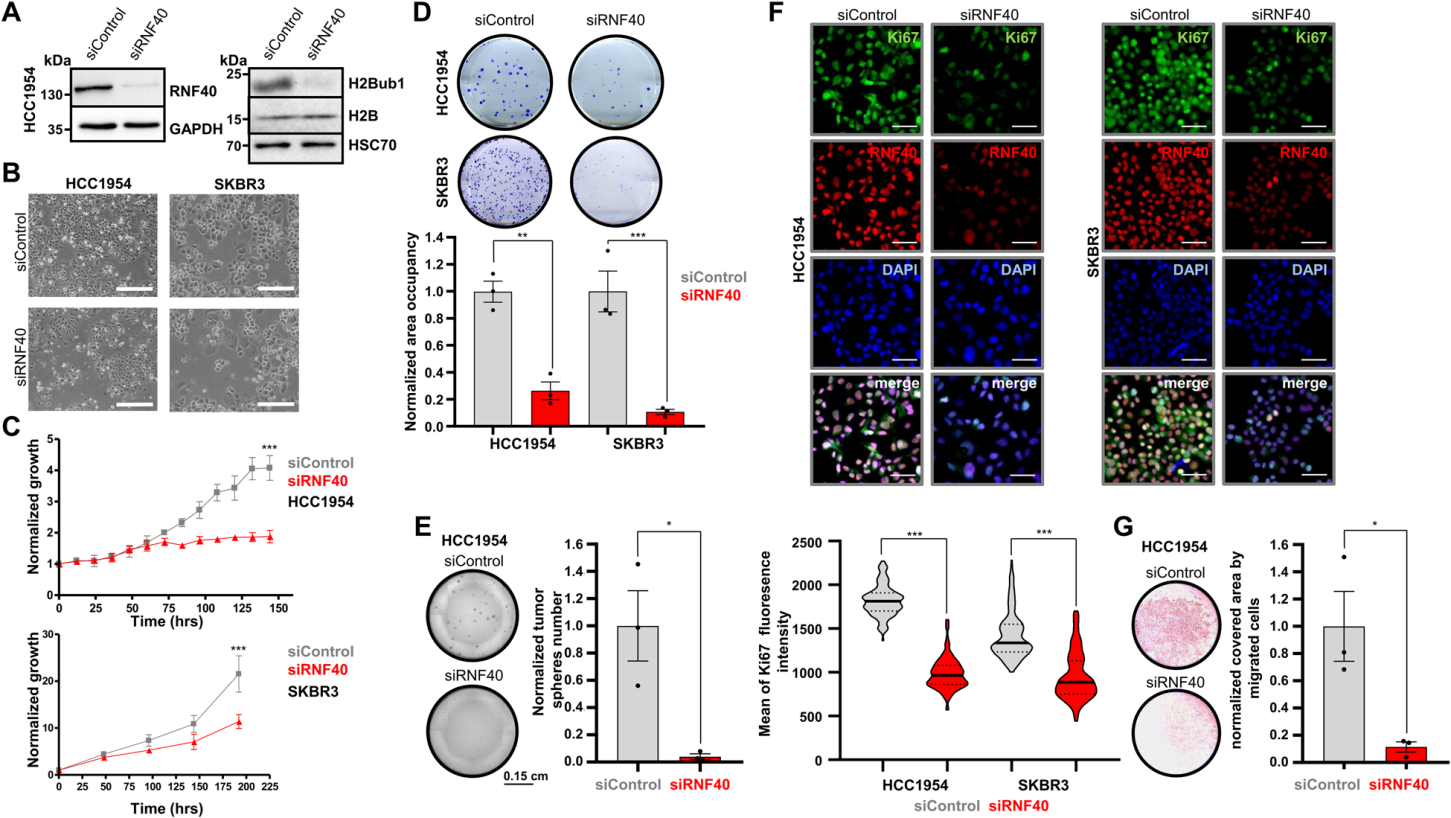
RNF40 loss impairs oncogenic properties of HER2-BC cells in vitro. **A** Western blot validation of RNF40 knockdown efficiency and decreased H2Bub1 levels in HCC1954 cells. **B** Representative bright-field pictures of control and RNF40 siRNA-transfected HCC1954 and SKBR3 cells. Scale bars (white): 500 µm. **C, D** Proliferation curves (**C**) and clonogenic assays (**D**) of control and RNF40-depleted HCC1954 and SKBR3 cells. Quantification of the occupied area in clonogenic assays is shown for both cell lines (**D**, lower panel). Student *t*-test. **E** Tumorsphere formation assay of control and RNF40-depleted HCC1954 cells (left panel). Quantification of the tumorsphere number normalized to the control condition (right panel). Student *t*-test. **F** Representative pictures from immunofluorescence detection of RNF40 and the proliferation marker Ki67 in control and RNF40-depleted HCC1954 and SKBR3 cells (upper panel). Scale bars = 60 μm. Quantification of the Ki67 immunofluorescence intensity of single nuclei in control and RNF40-depleted HCC1954 and SKBR3 cells (lower panel). The median intensity values of the respective groups are provided as green bars. Mann–Whitney test. **G** Transwell migration assay of control and RNF40-depleted HCC1954 cells with representative results (left panel) and the corresponding quantification (right panel). *p < 0.05, **p < 0.01, ***p < 0.005. All experiments were performed in biological triplicates. Error bars: SEM.

We next sought to investigate the underlying molecular mechanisms determining the dependence of HER2^+^-BC on RNF40. For this purpose, we selected two different human HER2^+^-BC cell lines (HCC1954, SKBR3) and assessed different parameters related to their tumorigenic properties following siRNA-mediated RNF40 knockdown. RNF40 depletion and concomitant loss of H2Bub1 in both cell lines (Fig. 2A, Fig. S2A–B) resulted in reduced cellular confluency compared to control transfected cells (Fig. 2B). Furthermore, growth kinetics (Fig. 2C and supplementary video), clonogenic capacity (Fig. 2D) and tumorsphere formation (Fig. 2E, Fig. S2C) were strongly impaired upon RNF40 loss in HER2^+^-BC cell lines. In support of these results, an analysis of gene essentiality data from the DepMap portal (https://depmap.org/), revealed that HER2^+^-BC cell lines are strongly dependent on *RNF40* expression (Fig. S2D). Consistently, the levels of the proliferation marker Ki67 were markedly reduced in both HER2^+^-BC cell lines upon RNF40 depletion (Fig. 2F). Finally, we also tested the migration potential of HCC1954 cells upon RNF40 depletion in a transwell migration (Fig. 2G) and a gap closure assay (Fig. S2E). Both approaches revealed impaired cellular motility upon RNF40 loss. Together with our in vivo observations, these findings support that *RNF40* expression is essential for maintaining tumorigenic properties of HER2^+^-BC cells in vitro and in vivo.

### RNF40 regulates actin-cytoskeleton-related genes in HER2^+^-BC

**Fig. 3:**
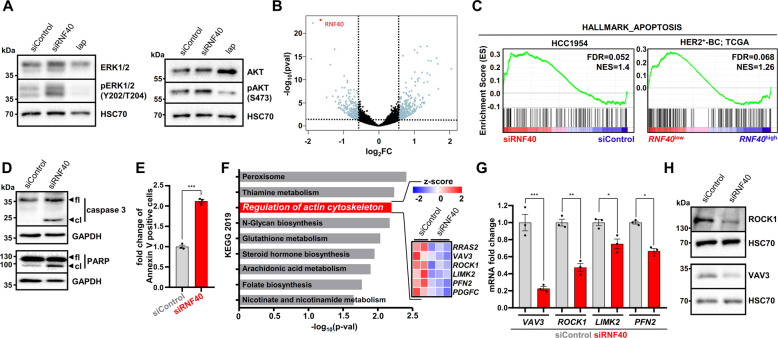
RNF40 loss increases apoptosis and impairs the expression of key components of the actin regulatory pathway in HER2+-BC. **A** Western blot analysis of the total and phosphorylated forms of ERK1/2 and AKT in control and RNF40-depleted HCC1954 cells. 1 µM Lapatinib (lap) was applied for 12 h as a positive control. **B** Volcano plot displaying gene expression changes occurring in HCC1954 cells upon RNF40 depletion and measured by mRNA sequencing. **C** Gene Set Enrichment Analysis (GSEA) of the mRNA-sequencing data identified the “HALLMARK_APOPTOSIS” geneset as being significantly enriched in the RNF40-depleted HCC1954 cells and in *RNF40*^low^-expressing HER2^+^-BC patients (TCGA). NES normalized enrichment score. **D** Western blot analysis showing that the markers of apoptosis, the cleaved forms of caspase 3 and PARP, are increased in RNF40-depleted HCC1954 cells compared to the control condition. **E** Annexin-V-based flow cytometric analysis of control and RNF40-depleted HCC1954 cells. **F** Pathway enrichment analysis (Enrichr tool; https://amp.pharm.mssm.edu/Enrichr/) showing that genes significantly downregulated upon RNF40 knockdown (log2FC ≤ −0.6, *p* < 0.05) are enriched for the KEGG 2019 signature “Regulation of actin cytoskeleton”. A heatmap depicting the differential expression of genes extracted from this signature is shown in the right panel. **G**, **H** The identified signature was validated via qRT-PCR (**G**) and western blot (**H**) for selected genes in HCC1954 cells. Student *t*-test. *p < 0.05, **p < 0.01, ***p < 0.005. All experiments were performed in biological triplicates. Error bars: SEM.

To understand the underlying mechanism underlying the anti-prolferative effects of RNF40 loss, we tested whether the activity of the signaling cascade downstream of HER2 may be directly affected by RNF40 loss. However, while the HER2 inhibitor lapatinib (lap) significantly blocked ERK and AKT phosphorylation, we could not observe any impairment of the two pathways following RNF40 depletion in either HCC1954 or SKBR3 cells, suggesting that the observed RNF40-dependent effects are not due to alterations in HER2 signaling (Fig. 3A, Fig. S3A). Therefore, given the transcriptional regulatory role of H2Bub1, we performed mRNA-sequencing (mRNA-seq) analyses of HCC1954 cells following *RNF40* depletion and identified 360 up- and 324 downregulated genes (|log2 fold change | >0.6; *p* < 0.05) (Fig. 3B). Consistent with our previous findings in colorectal cancer^19^, Gene Set Enrichment Analysis (GSEA) identified a significant enrichment of the “HALLMARK_APOPTOSIS” signature, potentially explaining the reduced oncogenic properties (Fig. 3C, Fig. S3B). In accordance, stratifying HER2^+^-BC patients from The Cancer Genome Atlas (https://portal.gdc.cancer.gov/) according to *RNF40* expression levels using publically available mRNA-seq data, we observed that *RNF40*^low^ tumors are also enriched for the same “HALLMARK_APOPTOSIS” geneset (Fig. 3C, Fig. S3C). Increased apoptosis was confirmed by microscopic time-lapse analyses (see supplementary videos), higher levels of cleaved caspase 3 and cleaved PARP in Western blot (Fig. 3D, Fig. S3D) and an increase in Annexin-V-positive cells (Fig. 3E). Given the fact that RNF40 depletion resulted in decreased cell number and cell migration, we performed additional gene ontology analyses and identified an actin-cytoskeleton regulatory pathway signature as being downregulated following RNF40 depletion (log2FC ≤ −0.6, *p* < 0.05) (Fig. 3F and S3E). The downregulation of Vav Guanine Nucleotide Exchange Factor 3 (*VAV3)*, Rho-Associated Coiled-Coil Containing Protein Kinase 1 (*ROCK1)*, LIM Domain Kinase 2 (*LIMK2),* and Profilin 2 (*PFN2)*, which directly control filamentous actin dynamics, was confirmed in both siRNF40-depleted cell lines at the mRNA (Fig. 3G and Fig. S3F) and protein levels (ROCK1, VAV3; Fig. 3H, Fig. S3G).

**Fig. 4:**
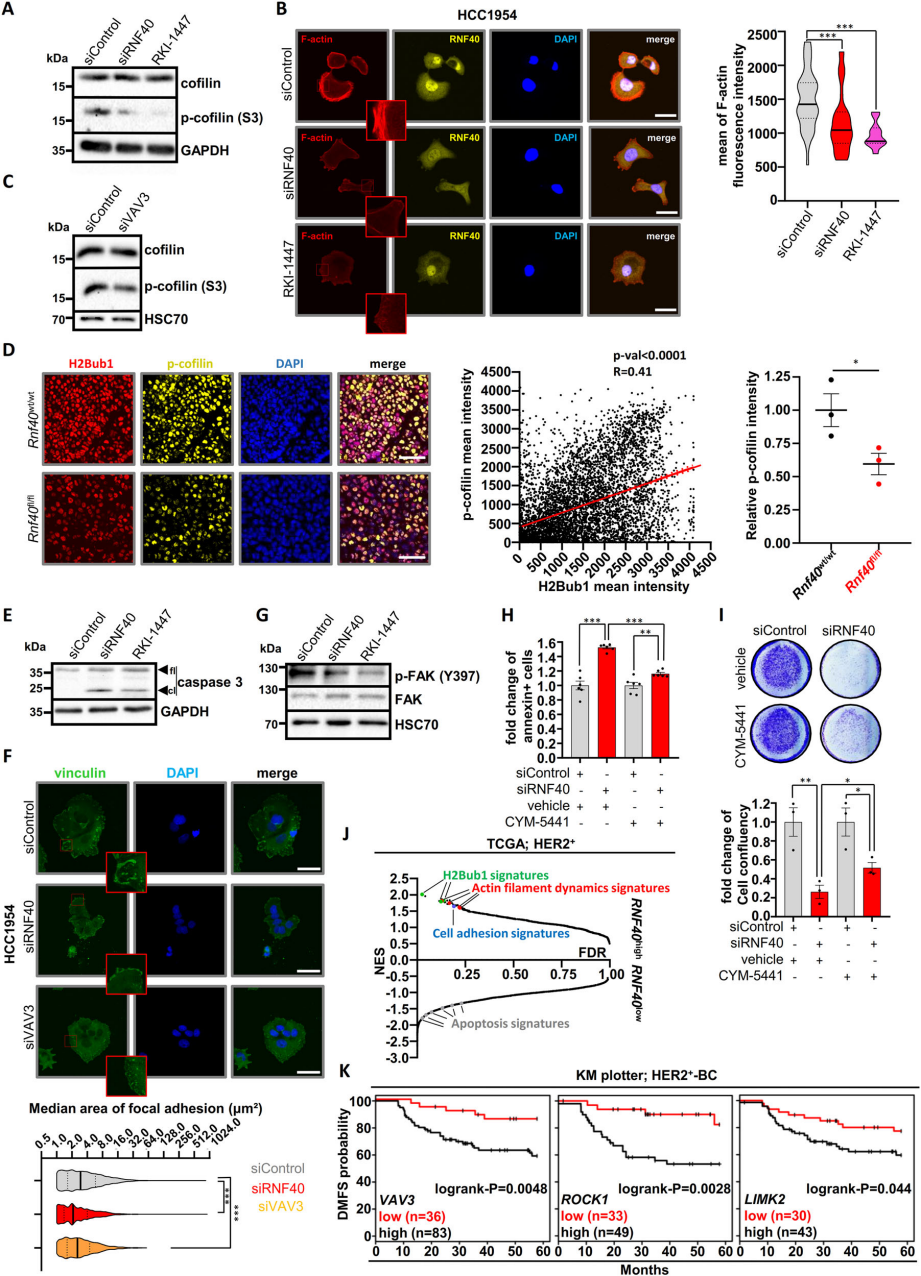
RNF40 controls the actin regulatory pathway to sustain the viability of HER2^+^-BC cells in vitro and in vivo. **A** Western blot analysis showing a reduction of phosphorylated cofilin (p-cofilin) upon RNF40 knockdown and ROCK inhibitor treatment (RKI-1447, 16 µM) in HCC1954 cells. **B** Representative pictures of immunofluorescence staining for F-actin in control, RNF40-depleted and RKI-1447-treated (16 μΜ) HCC1954 cells (right panel). Quantification of F-actin intensity in the respective conditions (left panel). Scale bars (white) = 50 μm. ANOVA one-way (Kruskal–Wallis test). **C** Western blot analysis showing a reduction of phosphorylated cofilin in VAV3-depleted HCC1954 cells. **D** Representative pictures of p-cofilin detected by immunofluorescence (left panel), scatter plot showing a correlation of H2Bub1 and p-cofilin intensity in the single tumor cells (middle panel) and quantification of the relative p-cofilin intensity in *Rnf40*^wt/wt^ and *Rnf40*^fl/fl^ tumors (right panel, Student *t*-test). **E** Western blot analysis assessing cleaved caspase 3 levels (cl cleaved, fl full length) in control, RNF40-depleted and RKI-1447-treated HCC1954 cells. **F** Representative immunofluorescence pictures of vinculin in control, RNF40-depleted and VAV3-depleted HCC1954 cells (right panel). Bar graph displaying the median focal adhesion area in the respective conditions (left panel). Scale bars = 50 μm. ANOVA one-way (Kruskal–Wallis test). **G** Western blot analysis assessing phosphorylated and total FAK levels of control, RNF40-depleted and RKI-1447-treated (16 μΜ) HCC1954 cells. **H, I** Annexin V assay (**H**) and proliferation assay (**I**) of control and RNF40-depleted HCC1954 cells with and without the S1PR_3_ agonist CYM-5441 (10 μΜ). Quantification of cell confluency (lower panel). Student *t*-test. **J** Graphical representation of all gene sets (C5_GO_Biological_Process) enriched in *RNF40*^high^- and *RNF40*^low^-expressing HER2^+^-BC biopsies in GSEA analyses. Normalized gene expression data were retrieved from the TCGA portal (https://portal.gdc.cancer.gov/). **K** Distant Metastasis-Free Survival (DMFS) based on the expression of *VAV3*, *ROCK1*, and *LIMK2* in HER2^+^-BC patients. Data were retrieved from the online tool KM plotter (kmplot.com). *p < 0.05, **p < 0.01, ***p < 0.005. All experiments were performed in biological triplicates. Error bars: SEM.

Phosphorylation of the cofilin protein by LIMK downstream of ROCK1 plays an important role in controlling actin-cytoskeleton dynamics^39^. In its active unphosphorylated form, cofilin destabilizes filamentous actin (F-actin) and leads to actin depolymerization. Interestingly, cofilin phosphorylation (p-cofilin) levels were strongly reduced in RNF40-depleted HCC1954 cells (Fig. 4A). Phalloidin staining confirmed impaired F-actin formation upon RNF40 depletion in HCC1954 (Fig. 4B) and SKBR3 cells (Fig. S4A) and these effects could be phenocopied by ROCK1 inhibition (Fig. 4A, B) and VAV3 knockdown (Fig. 4C). To exclude a bi-directional regulation between RNF40 and ROCK1, we verified that RNF40 protein levels remained unchanged upon ROCK1 inhibition (Fig. S4B). Importantly, cofilin phosphorylation was also markedly decreased in murine *Rnf40*^fl/fl^ tumors compared to wild type counterparts and significantly associated with H2Bub1 levels (Fig. 4D). Together, these data confirm the in vitro and in vivo importance of the RNF40/H2Bub1 axis in controlling actin cytoskeletal dynamics in HER2^+^-BC.

The role of the ROCK1 pathway is not limited to the control of actin cytoskeletal dynamics, but also plays a central role in suppressing apoptosis, potentiating cell survival and is significantly associated with poor prognosis in HER2^+^-BC patients^40–44^. Thus, we hypothesized that the dysregulation of the ROCK1-dependent actin regulatory pathway may be responsible for the apoptotic phenotype induced by RNF40 loss. Indeed, inhibition of ROCK1 by RKI-1447 led to impaired HCC1954 cell proliferation (Fig. S4C) and increased caspase 3 cleavage (Fig. 4E).

Cell-to-substrate focal adhesion complexes are tightly coupled to the actin cytoskeleton and significantly contribute to preserving antiapoptotic pathways via the Focal Adhesion Kinase (FAK)^42,45^. Thus, we hypothesized that RNF40 depletion may impair focal adhesion signaling by interfering with actin dynamics. To test this hypothesis, we examined if RNF40 loss influences the size of focal adhesions by performing immunofluorescence staining for vinculin, one of the molecules bridging focal adhesion complexes and F-actin. Indeed, the focal adhesion area was substantially decreased upon RNF40 depletion and these effects were phenocopied by VAV3 depletion (Fig. 4F). Furthermore, both RNF40 depletion and ROCK inhibition reduced the levels of active phosphorylated FAK (p-FAK) (Fig. 4G). Moreover, consistent with these findings, direct inhibition of FAK led to a significant decrease of HCC1954 cell growth (Fig. S4D). Together, our data suggest that RNF40 sustains the tumorigenic phenotype of HER2^+^-BC cells by maintaining actin dynamics and FAK-activity in a ROCK1-dependent manner.

To confirm a role for the actin regulatory pathway in the impaired tumorigenic phenotype of HCC1954 cells upon RNF40 loss, we examined the effects of restoring this signaling cascade. For this purpose, we treated HCC1954 cells with an allosteric sphingosine 1-phosphate receptor-3 agonist (CYM-5441), which was shown to activate actin polymerization as well as increase cancer stem cell expansion in BC^46,47^. Treatment of RNF40-depleted HCC1954 cells with CYM-5441 significantly rescued apoptosis as measured by Annexin V staining (Fig. 4H) and caspase 3/7 activity (Fig. S4E). Additionally, treatment with either CYM-5441 (Fig. 4I) or lysophosphatidic acid (Fig. S4F), which has also been shown to activate this pathway^48^, partially rescued the impaired proliferation of HCC1954 cells following RNF40 depletion. Notably, this rescue was prevented by treatment with RKI-1447 (Fig. S4G-H), confirming that partial restoration of the actin regulatory pathway is central to the observed patial rescue.

Finally, to verify our findings in human HER2^+^-BC samples, we performed GSEA analyses on publically available mRNA-seq data from The Cancer Genome Atlas (TCGA; https://portal.gdc.cancer.gov/) stratifying patients based on *RNF40* expression. In addition to H2Bub1-related gene signatures, *RNF40*^high^ tumors were also significantly enriched for gene sets characteristic for actin filament dynamics and cell adhesion. Similarly, *RNF40*^low^ tumors were enriched for apoptotic gene signatures (Fig. 4J, Fig. S4I, J). In accordance, like *RNF40* (Fig. S1B), higher *VAV3*, *ROCK1* and *LIMK2* expression is associated with distant metastasis and poor survival outcome in HER2^+^-BC patients (Fig. 4K).

Collectively, these findings establish the RNF40/H2Bub1 axis as an important regulator of HER2^+^-BC cell viability by controlling actin regulatory dynamics and focal adhesion signaling via the VAV3-ROCK1-LIMK2-PFN2 cascade.

### RNF40 regulates the VAV3-ROCK-LIMK2-PFN2 axis through H2Bub1-H3K4me3 trans-histone crosstalk

**Fig. 5:**
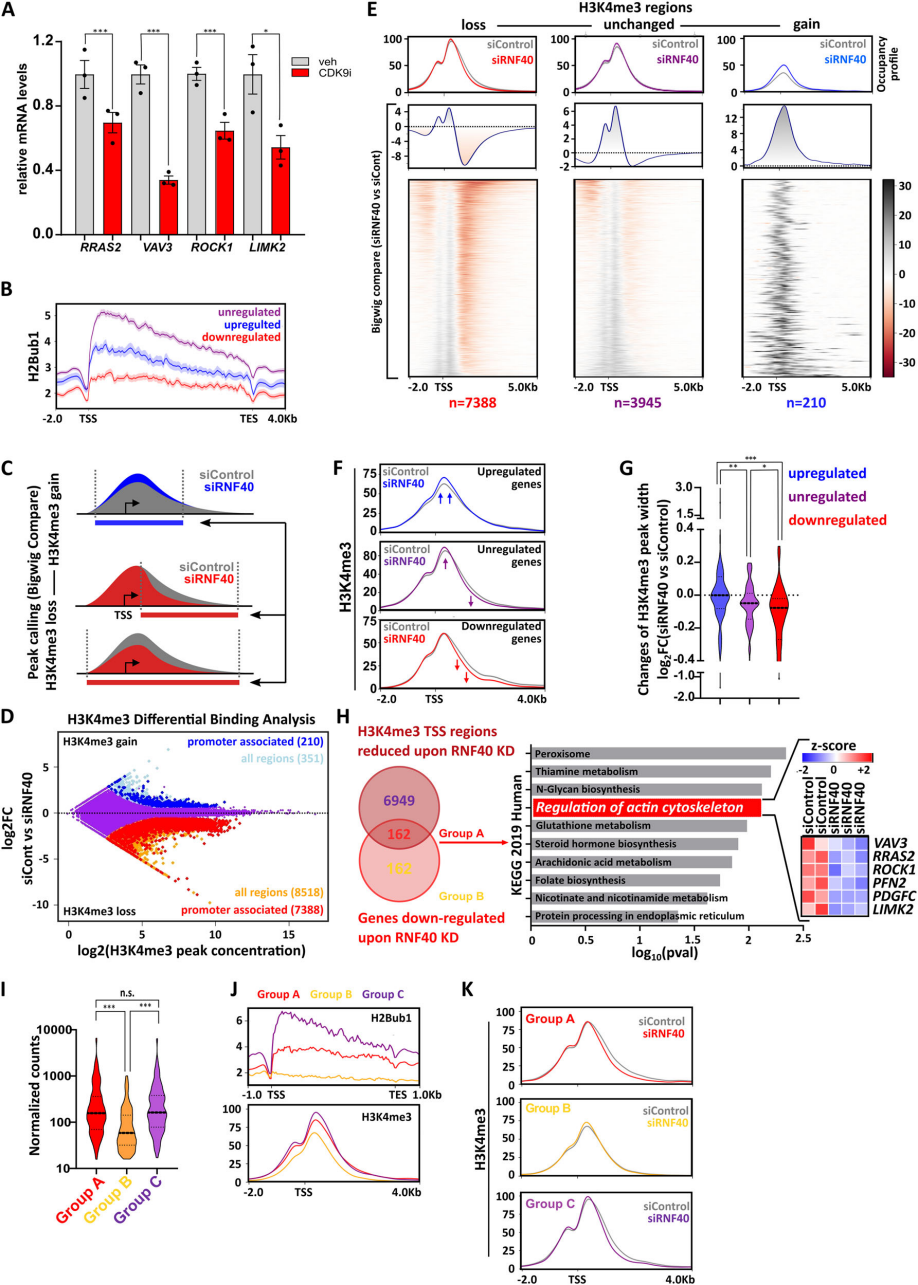
RNF40 regulates gene expression of important members of the RHO-ROCK axis in an H2Bub1/H3K4me3-dependent manner. **A** Expression levels of RNF40-dependent actin regulatory genes in DMSO- and CDK9i-treated (BAY-1251152) HCC1954 cells (120 nM, 6 h) assessed by qRT-PCR. Student *t*-test. **B** H2Bub1 occupancy profiles at gene body of down-, up-, and unregulated genes upon RNF40 depletion (regulated genes |log2FC | ≥0.6, *p* < 0.05, unregulated genes |log2FC | ≤0.1, *p* > 0.95). **C** Schematic workflow showing the procedure utilized to identify regions losing or gaining H3K4me3 occupancy upon RNF40 depletion. **D** Differential Binding Analysis results showing H3K4me3 regulated (|log2FC | ≥0.7, FDR < 0.05) and unregulated regions (in purple). **E** Heatmaps and respective aggregate profiles depicting changes of H3K4me3 occupancy in the identified gained (log2FC ≥ 0.7, FDR < 0.05, peak concentration≥6.2), lost (log2FC ≤ −0.7, FDR < 0.05, peak concentration≥6.2) or unregulated (|log2FC | ≤0.2, FDR > 0.1, peak concentration≥6.2) regions upon RNF40 depletion based on the DiffBind analysis results in **C**. **F** Aggregate plots showing changes of H3K4me3 occupancy at TSS-associated regions of genes identified in RNA-seq analysis as robustly down-, up- (|log2FC | ≥0.8, *p* < 0.05), and unregulated (|log2FC | ≤0.1, *p* > 0.95) following RNF40 depletion. **G** Quantification of changes in H3K4me3 peak width upon RNF40 depletion in regulated and unregulated genes. ANOVA one-way (Kruskal–Wallis test). **H** Left panel: classification of genes influenced by RNF40 depletion into Group A (simultaneous downregulation and H3K4me3 loss at TSS region), Group B (downregulation without H3K4me3 loss). Right Panel: Group A genes were analyzed for pathway enrichment using the online Enrichr web tool (https://amp.pharm.mssm.edu/Enrichr3/). **I** Violin plot providing the median of normalized counts of the three gene groups (Group A, B, and C). ANOVA one-way (Kruskal–Wallis test). **J** Aggregate plots of H2Bub1 and H3K4me3 at basal state in Group A, B, and C genes. **K** Changes in H3K4me3 occupancy at TSS-regions of group A, B, and C genes. H3K4me3 and H2Bub1 ChIP-seq experiments were performed in biological triplicates or duplicates, respectively. *p < 0.05, **p < 0.01, ***p < 0.005.

Cyclin-dependent kinase 9 (CDK9) is a critical upstream regulator of H2B monoubiquitination that functions to recruit the WAC/RNF20/RNF40 complex by phosphorylating the carboxy-terminal domain (CTD) of RNA Polll, thereby directly coupling H2B monoubiquitination to transcriptional elongation^7,24^. Thus, we hypothesized that inhibition of CDK9 would phenocopy the effects of RNF40 loss without impairing RNF40 protein expression levels, thereby providing further support for the role of H2Bub1 in the observed effects. Indeed, treatment of HCC1954 cells with a CDK9-specific inhibitor (BAY-1251152; CDK9i, 120 nM) led to a pronounced reduction of H2Bub1 and impaired the expression of all RNF40-dependent actin regulatory genes (Fig. 5A and S5A). Furthermore, CDK9 inhibition phenocopied the proliferative defects and the F-actin impairment of RNF40-depleted cells, without impairing RNF40 protein expression (Fig. S5B-C). Conversely, overexpression of CDK9 in HCC1954 cells significantly increased the expression of RNF40-dependent actin regulatory genes (Fig. S5D). Together, these results support a central function of RNF40-mediated H2B monoubiquitination in regulating the expression of central actin regulatory factors.

Previous studies described a crosstalk between H2Bub1 and H3K4 trimethylation (H3K4me3) both in yeast and human systems^26,29,30^. Moreover, we recently demonstrated that RNF40-mediated H2B monoubiquitination governs the broadening of H3K4me3 from the transcriptional start site (TSS) into the transcribed region to facilitate transcriptional elongation of many moderately H2Bub1-marked genes in mouse embryo fibroblasts (MEFs) and osteoblasts^10,22^. To examine if RNF40 controls the expression of genes of the actin regulatory network by modulating H2Bub1 and H3K4me3 levels, we performed chromatin immunoprecipitation sequencing (ChIP-seq) analyses (Fig. S5E). Strikingly, consistent with our previous findings^22^, RNF40-dependent genes showed lower levels of H2Bub1 occupancy compared to unregulated genes or genes upregulated following RNF40 depletion (Fig. 5B). Given our previous finding that H3K4me3 “peak narrowing” is a distinct epigenetic feature involved in the regulation of RNF40-dependent genes, we identified regions with an increase or a global or partial (3’ narrowing) decrease of H3K4me3 occupancy upon RNF40 silencing (Fig. 5C). The identified regions were then used for differential binding (DiffBind) analyses. Interestingly, the majority of the regions influenced by RNF40 depletion markedly lost H3K4me3 (8518 regions), whereas only a few regions gained H3K4me3 occupancy (351 regions) (Fig. 5D and Fig. 5E). Most of the regions showing decreased H3K4me3 (Fig. 5D) were located near TSS regions (Fig. S5F). Moreover, regions displaying no changes in H3K4me3 occupancy displayed only a mild peak narrowing, while regions displaying a significant loss of H3K4me3 occupancy exhibit a stronger peak narrowing upon RNF40 depletion (Fig. 5E and S5G). Importantly, consistent with our gene expression analyses, TSS-associated regions displaying decreased H3K4me3 occupancy following RNF40 depletion included genes associated with the actin regulatory pathway signature (Fig. S5H).

We next investigated changes of H3K4me3 occupancy at TSS of robustly down-, up-, and unregulated genes under control or RNF40-depleted conditions. Genes downregulated upon RNF40 silencing displayed the most pronounced decrease in H3K4me3 occupancy in the gene body (the 3’ end of the peak) compared to unregulated or upregulated genes (Fig. 5F-G). Importantly, a significant fraction of downregulated genes (162 out of 324, log2FC ≤ −0.6, *p* < 0.05) showed a concomitant decrease in H3K4me3 near the TSS (“Group A” in Fig. 5H). Moreover, this group was enriched for genes involved in controlling actin dynamics. We validated the decrease in H3K4me3 spreading into the gene body of the *ROCK1*, *LIMK2* and *VAV3* genes by ChIP-qPCR (Fig. S5I).

As a control, we identified a group of genes with a similar size, similar expression range and comparable H3K4me3 peak width as Group A (Group C), whose expression was not affected by RNF40 knockdown but was characterized by a reduction of H3K4me3 occupancy (Fig. 5I and S6A). We tested the sensitivity of this control group to H2Bub1 loss by verifying the regulation of randomly selected Group C members upon CDK9i treatment. Strikingly, none of the four tested genes were found to be regulated, confirming the validity of our approach (Fig. S6B). Under control conditions, Group A genes harbored lower H2Bub1 levels than Group C genes, but comparable H3K4me3 levels and peak height (Fig. 5J and Fig. S6C). However, Group A genes presented a more profound H3K4me3 peak narrowing upon RNF40 depletion compared to Group C (Fig. 5K and Fig. S6D). The 162 genes found to be downregulated at the mRNA level, but not showing any H3K4me3 loss (Group B), displayed overall lower expression values, smaller H3K4me3 regions and displayed only negligible levels of H2Bub1 across their gene body (Fig. 5H–K, Fig. S6A). We, therefore, concluded that the genes within Group B may be indirect downstream targets of RNF40-dependent regulation. Together, these findings support the hypothesis that the actin regulatory gene network is dependent on direct epigenetic regulation by RNF40 through modulation of H2Bub1 and a trans-histone crosstalk with H3K4me3 in HER2^+^-BC cells.

To further investigate the importance of H3K4me3 driving the expression of the actin regulatory genes, we treated HCC1954 cells with OICR9429, which impairs COMPASS-dependent H3K4 methylation by inhibiting WDR5, a common subunit of the SETD1A/B- and MLL1/2-containing methyltransferase complexes^49^. Consistent with our hypothesis, all identified RNF40-dependent actin regulatory genes were downregulated upon treatment, confirming the central role of H2Bub1-dependent H3K4 trimethylation in activating these genes (Fig. S6E). Moreover, given the close connection between H3K4me3 spreading and transcriptional elongation at specific genes^31^, we hypothesized that interfering with the negative elongation factor (NELF) might rescue the expression of the actin regulatory genes upon RNF40 loss. Consistently, depletion of NELF-E partially restored the expression of all RNF40-dependent actin regulatory genes in the absence of RNF40 (Fig. S6F). This further supports our findings with CDK9 inhibition and further suggests an intimate connection between RNA Pol II elongation, H2Bub1, and H3K4me3 in controlling genes in the actin-cytoskeleton network.

To finally characterize the epigenetic landscape distinguishing Group A and C, and which may further help to explain the RNF40-dependency of Group A genes, we analyzed ChIP-seq data for several other histone modifications in HCC1954 cells^33,34^. These analyses revealed that the occupancy of the active histone marks H3K27ac and H3K9ac was slightly higher near the TSS of Group C genes in comparison to Group A, while the elongation-associated modifications, H3K36me3 and H3K79me2, were dramatically higher in the gene body of Group C. Accordingly, RNA Pol II occupancy was also higher on genes in Group C compared to the other groups (Fig. S6G). Together, when compared to the genes within Groups A and B, genes within Group C display a more pronounced occupancy of epigenetic marks associated with active gene transcription^50^. Thus, these additional epigenetic modifications may help to compensate for the loss of H2Bub1 following RNF40 depletion, whereas lower levels of these active marks on Group A genes may render them to be more sensitive to changes in H2Bub1/H3K4me3 occupancy.

In summary, we conclude that RNF40 is a major epigenetic regulator of the actin regulatory gene network in HER2^+^-BC cells via H2B monoubiquitination and the downstream *trans*-histone control of H3K4me3 occupancy in the transcribed region.

## Discussion

H2B monoubiquitination has previously been reported to serve a tumor-suppressive function with its levels gradually decreasing during cancer progression. Interestingly, the role of RNF20, a subunit of the obligate heterodimeric RNF20/RNF40 E3 ubiquitin ligase complex catalyzing the deposition of H2Bub1, is more contradictory and has been reported to exert opposing functions depending on cancer type or subtype^16,17^. To date, only few studies examined RNF40 expression in cancer. Upon examination of a cohort of both primary BC and brain metastases, we identified the loss of RNF40 expression and H2Bub1 as rare events in primary and metastatic HER2^+^-BC lesions. Publically available datasets corroborate our results, showing only a very low rate of genetic alterations (<1%) causing loss of RNF40 function in BC (cbioportal.org, data not shown). Interestingly, the same datasets report a much higher frequency of *RNF40* locus amplification in malignancies of the breast (4–6%) accompanied by increased *RNF40* expression levels in tumors compared to normal tissues (TCGA dataset). Additionally, high expression levels of *RNF40* were associated with an unfavorable outcome in HER2^+^-BC patients. Finally, the genetic model for *Rnf40* loss in endogenous HER2-driven mammary carcinomas used in this study supported the human patient data, arguing for a tumor-supporting role for RNF40 in HER2-dependent BC. Together, our data do not support a general tumor-suppressive function of RNF40 and H2Bub1.

Upon investigating the transcriptional and molecular epigenetic mechanisms rendering HER2^+^-BC cells critically dependent upon RNF40, we observed that loss of RNF40 had a profound impact on the pattern of occupancy of H3K4me3 leading to a significant “peak narrowing” in the transcribed region downstream of the TSS on regulated genes. The crosstalk between H2Bub1 and H3K4me3 has been intensively studied in the past and has been attributed to the trans-regulation of the histone methyltransferase activity of the COMPASS family of H3K4 methyltransferases by H2Bub1^7,10,22,24^. Our previous work revealed that RNF40 promotes the expression of a specific subset of genes displaying a high elongation rate via modulation of H3K4me3 peak broadening. Our new integrated datasets in HER2^+^-BC not only support a role for RNF40 in maintaining transcriptional elongation-associated spreading of H3K4me3, but also show that these genes display a less pronounced accumulation of various activating epigenetic marks compared to RNF40-independent genes. Interestingly, RNF40-dependent genes also displayed lower H3K79me2 levels, another histone mark that was shown to function downstream of H2Bub1 to epigenetically regulate gene expression, implying that an additional epigenetic layer helps to control the transcriptional output of RNF40/H2Bub1-independent genes^28^. We therefore hypothesized that this specific group of genes is rendered particularly sensitive to H2Bub1 loss upon RNF40 depletion due to their overall less active chromatin status.

Strikingly, many genes identified in this study as being RNF40-dependent are well-known effectors of the actin regulatory pathway. In addition to the reported implication of RNF40 in the DNA damage response^51^, replication stress^14^, microtubule spindle organization^52^, inflammation^18^, and regulation of hormone receptor activity^12,20^, the discovery that the maintenance of actin dynamics critically depends upon RNF40 in HER2^+^-BC is both new and of significant interest. Notably, HER2^+^-BC cells were previously shown to heavily rely on intact actin dynamics for cancer cell viability, motility and metastasis^43,53^. Importantly, we specifically identified *VAV3*, *ROCK1*, *LIMK2*, and *PFN2* as RNF40-dependent genes and confirmed the functional consequence of their impaired expression, which resulted in decreased cofilin phosphorylation both in vitro and in vivo, and decreased F-actin abundance and impaired actin dynamics. Importantly, we identified the ROCK1 kinase as a central RNF40-regulated factor controlling the actin regulatory pathway. Consistently, inhibition of ROCK activity using the specific inhibitor RKI-1447 phenocopied the impaired tumorigenic phenotype caused by RNF40 loss. Interestingly, activation of the actin-cytoskeleton signaling pathway by treating RNF40-depleted cells with an S1PR_3_ agonist partially rescued these effects. Therefore, these data strongly suggest that the imbalance in the control of actin dynamics in RNF40-depleted cells is largely dependent on the loss of ROCK1 activity.

In addition to the central role of actin-cytoskeleton dynamics in controlling cellular migration, ROCK1, F-actin and the focal adhesion kinase signaling pathway also have critical functions in suppressing apoptosis and are strongly associated with BC progression including HER2^+^-BC^41,44,54–57^. While we previously identified a role for RNF40 in suppressing apoptosis in colorectal cancer cells via expression of antiapoptotic members of the BCL2 family of proteins^19^, our current results suggest that RNF40 supports HER2^+^-BC tumor viability and tumorigenic features in a distinct manner via maintenance of ROCK-dependent focal adhesion kinase signaling. Our data suggest that RNF40-driven H2B monoubiquitination plays a decisive, context-specific function in HER2^+^-BC by controlling the actin regulatory circuit and downstream signaling to maintain antiapoptotic signaling and promote cellular migration in cancer cells. It is therefore attractive to speculate that simultaneous inhibition of the RNF20/RNF40 E3 ubiquitin ligase activity, or upstream regulatory components such as CDK9, together with inhibition of either ROCK1 or FAK might provide synergistic effects in the treatment of HER2^+^-BC.

**Fig. 6:**
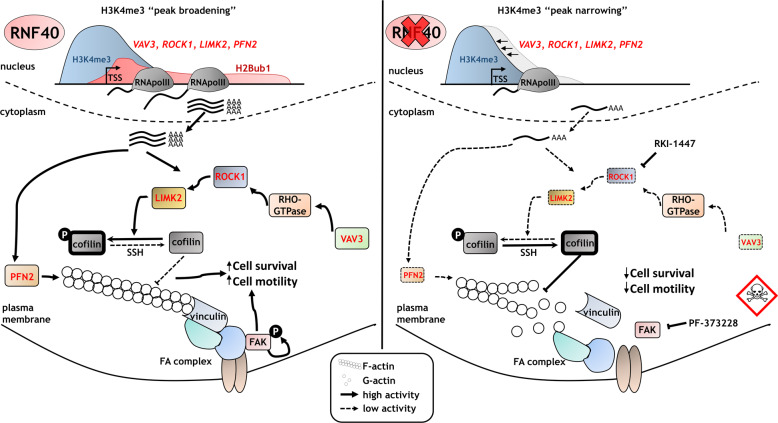
RNF40 has a tumor-supportive function in HER2-driven mammary carcinoma by controlling the RHO/ROCK-dependent actin regulatory axis. RNF40-driven H2B monoubiquitination is essential for transcriptional activation of the RHO/ROCK/LIMK pathway components and proper actin polymerization via trans-histone crosstalk of histone 3 lysine 4 trimethylation (H3K4me3). Loss of RNF40 impairs the H2Bub1-H3K4me3 trans-histone crosstalk, inducing H3K4me3 peak narrowing. Consequently, critical modulators of the actin dynamics are downregulated, impairing the formation of F-actin and focal adhesions complexes (FA) and ultimately reducing pro-survival signaling in HER2^+^-BC cells.

Together, our data support a context-dependent role of RNF40 and H2B monoubiquitination in breast carcinogenesis and suggest that the RNF20/RNF40 E3 ubiquitin ligase and/or its upstream regulators or downstream targets may serve as attractive targets for the development of new anticancer strategies in HER2^+^-BC (Fig. 6).

## Supplementary information

Supplementary Text and Tables

Supplementary Figure S1

Supplementary Figure S2

Supplementary Figure S3

Supplementary Figure S4

Supplementary Figure S5

Supplementary Figure S6

Related Manuscript File

Related Manuscript File
