# Supplementary material for: The histone H2B ubiquitin ligase RNF40 is required for HER2-driven mammary tumorigenesis
Source: Cell Death Dis. 2020 Oct 17;11(10):873. doi: 10.1038/s41419-020-03081-w (PMC7568723; doi:10.1038/s41419-020-03081-w)
Supplement: Supplementary file 1 — Supplementary Text and Tables [file 41419_2020_3081_MOESM1_ESM.docx]

Supplementary Tables

Table S1: cell lines used in this study.

| **Human cell line** | **HCC1954** | **SKBR3** | **BT474** |
| --- | --- | --- | --- |
| **Tissue of origin** | 43 years old, female, mammary gland, adenocarcinoma | 61 years old, female, ductal carcinoma | 60 years adult, female, ductal carcinoma |
| **Morphology and growth properties** | epithelial, monolayer, adherent | epithelial, monolayer,  adherent | epithelial, patchy |
| **Supplier** | ATCC | ATCC | ATCC |
| **Recommended medium** | RPMI 1640. GlutaMAX^TM^ (Thermofisher Scientific) | DMEM/F12-Dulbecco’s Modified Eagle’s Medium: Nutrient Mixture F-12 (Thermofisher Scientific) | DMEM/F12-Dulbecco’s Modified Eagle’s Medium: Nutrient Mixture F-12 (Thermofisher Scientific) |
| **HER2 amplification status** | + | + | + |
| **ER/PR status** | -/- | -/- | -/+ |
| **p53 status** | mutated (Y136C) | mutated (R175H) | Mutated (E285K) |

Table S2: siRNAs used in this study.

| **Gene** | **siRNA** |
| --- | --- |
| Non-Τargeting #5 | UGGUUUACAUGUCGACUAA |
| *RNF40* | #1 GAGAUGCGCCACCUGAUUA |
|  | #2 GAUGCCAACUUUAAGCUAA |
|  | #3 GAUCAAGGCCAACCAGAUU |
|  | #4 CAACGAGUCUCUGCAAGUG |
| *VAV3* | #1 GCAGAGACCGAACUUAUUA |
|  | #2 GCAAAGCACAUCAAGAUUU |
|  | #3 AGACCGAACUUAUUAAUAG |
|  | #4 GUAUGCAGCCCUCGUGUAA |
| *NELFE* | #1 ATGGAGTCAGCAGATCAGTTCAAGAGACTGATCTGCTGACTCCATCTTTTT |
|  | #2 GATCAAAAAGATGGAGTCAGCAGATCAGTCTCTTGAACTGATCTGCTGACTCCATCA |

Table S3: qRT-PCR primers used in this study.

| **Gene name** | **Forward (5‘-3‘)** | **Reverse (5‘-3‘)** | **Species** | **Reference** |
| --- | --- | --- | --- | --- |
| ***RPLP0*** | GATTGGCTACCCAACTGTTG | CAGGGGCAGCAGCCACAAA | Human | (1) |
| ***Rplp0*** | GATTCGGGATATGCTGTTGG | GCCTGGAAGAAGGAGGTCTT | Mouse | This study |
| ***RNF40*** | AGTACAAGGCGCGGTTGA | GAAGCAGAAAACGTGGAAGC | Human | (2) |
| ***Rnf40*** | GGCCCAGCTGGATGAAACTGT | ACTGAGAGGGGCTCGAAACT | Mouse | This study |
| ***VAV3*** | CCAATGGACTGCGAAGAACT | GTAAAGGGGGTCCTTCATGC | Human | This study |
| ***ROCK1*** | CTGCAACTGGAACTCAACCA | GCCAACTGCATCTGAAGCTC | Human | This study |
| ***LIMK2*** | AGTCCCGGCTTACTTCACCT | CCCAAACTTCCCCCAGTAGT | Human | This study |
| ***PFN2*** | CCAAGAGTCAAACCGTTGGT | GGGCGTCTTTCAGAGCATTA | Human | This study |
| ***RRAS2*** | CGGATTATGATCCAACCATTG | TGTTCTCTCATGGCTCCAAAC | Human | This study |
| ***CTNNB1*** | TGGATACCTCCCAAGTCCTG | CTGAGCTCGAGTCATTGCAT | Human | This study |
| ***MYC*** | GGACGACGAGACCTTCAT | GCCGCTCCACATACAGTC | Human | This study |
| ***CFL1*** | GCAAGAAGGAGGATCTGGTG | CCTTGGAGCTGGCATAAATC | Human | This study |

Table S4: ChIP-qPCR primers used in this study.

| **Gene name** | **Forward (5‘-3‘)** | **Reverse (5‘-3‘)** | **Species** | **Reference** |
| --- | --- | --- | --- | --- |
| **Control primers** | | | | |
| ***GAPDH* (gene body)** | CCGGGAGAAGCTGAGTCATG | TTTGCGGTGGAAATGTCCTT | Human | (3) |
| ***HNRNPK* (TSS proximal region)** | TCCACGAGGTCCCTAGTTCC | GCCATTTCCCTGAGCGTGTA | Human | This study |
| **H3K4me3 narrowing assessment** | | | | |
| ***VAV3* (TSS proximal region)** | CCGGAGAGGAAACTTGTCAC | CCACCCTCCATCAGAGAAAA | Human | This study |
| ***ROCK1* (TSS proximal region)** | GGAGCTAAATCGCAAAAAGG | TTTGTTGAATGGCGATCGTA | Human | This study |
| ***LIMK2* (TSS proximal region)** | GAACCTTTAAGGGCCAGACC | AGGGGCAAGAAGATCAGGTT | Human | This study |

Table S5: List of primary antibodies.

|  | **WB (dilutions)** | **IHC**  **(dilutions)** | **IF**  **(dilutions)** | **ChIP**  **(dilutions)** | **cat.number/ company** |
| --- | --- | --- | --- | --- | --- |
| **HSC70** | (1:100) |  |  |  | sc-1050, Santa Cruz |
| **GAPDH** | (1:2000) |  |  |  | OTI2D9, Origene |
| **ACTIN** | (1:200) |  |  |  | sc-1616 (1-19), Santa Cruz |
| **HER2** |  | (1:500,EDTA buffer) |  |  | 2165, Cell Signaling |
| **RNF40** |  | (1:100,EDTA buffer) |  |  | ab191309, Abcam |
| **RNF40** | (1:1000) |  |  |  | D2R20, Cell Signaling |
| **RNF40** |  |  | (1:500) |  | R9029, Sigma Aldrich |
| **H2Bub1** | (1:50) | (1:100,EDTA buffer) |  |  | home-made |
| **H2Bub1** |  |  | (1:500,citric buffer) | 1 μg/sample | 5546, Cell Signaling |
| **H2B** | (1:500) |  |  |  | 07-371, Millipore |
| **Ki67** |  | (1:1000,citric buffer) |  |  | home-made (rabbit) |
| **H3K4me3** |  |  |  | 1 μg/sample | 003050, Diagenode |
| **PARP** | (1:500) |  |  |  | 9542, Cell Signaling |
| **caspase-3** | (1:500) |  |  |  | 9662, Cell Signaling |
| **p-ERK Y202/T204** | (1:200) |  |  |  | sc-7383, Santa Cruz |
| **ERK1** | (1:200) |  |  |  | sc-94, Santa Cruz |
| **p-AKT S473** | (1:500) |  |  |  | 736 E11, Cell Signaling |
| **AKT** | (1:500) |  |  |  | 9272, Cell Signaling |
| **ROCK1** | (1:200) |  |  |  | sc-17764, Santa Cruz |
| **VAV3** | (1:100) |  |  |  | CSB-PA006523-100, Cusabio |
| **p-cofilin S3** | (1:1000) |  | (1:100,citric buffer) |  | 77G2, Cell Signaling |
| **cofilin** | **(1:1000)** |  |  |  | D3F9, Cell Signaling |
| **vinculin** |  |  | **(1:200)** |  | V9131, Sigma Aldrich |

Table S6: List of secondary antibodies.

|  | **WB (Dilution)** | **IHC (Dilution)** | **IF**  **(Dilution)** | **Cat.number, company** |
| --- | --- | --- | --- | --- |
| **HRP-anti-rabbit IgG** | (1:10.000) |  |  | 211-032-171, Dianova |
| **HRP-anti-mouse IgG** | (1:10.000) |  |  | 115-035-174, Dianova |
| **Alexa 555-anti-rabbit IgG** |  |  | (1:400) | A-21429, Molecular Probes |
| **Alexa 488-anti-mouse IgG** |  |  | (1:400) | A-21202, Molecular Probes |
| **DyLight 649-anti-rabbit IgG** |  |  | (1:400) | 711-495-152, Dianova |
| **Alexa 555-anti-mouse IgG** |  |  | (1:400) | A-31570, Molecular Probes |
| **biotin-anti-rabbit IgG** |  | (1:200) |  | 711-065-152, Dianova |
| **biotin-anti-mouse IgG** |  | (1:200) |  | 711-065-150, Dianova |

Table S7: Average ChIP-seq library size.

| **sample** | **Average library size** | **Accession number** |
| --- | --- | --- |
| H3K4me3_siControl (replicate 1) | 326 bp |  |
| H3K4_siRNF40 (replicate 1) | 334 bp |  |
| H3K4me3_siControl (replicate 2) | 300 bp |  |
| H3K4_siRNF40 (replicate 2) | 330 bp |  |
| H3K4me3_siControl (replicate 3) | 394 bp |  |
| H3K4_siRNF40 (replicate 2) | 285 bp |  |
| H2Bub1_siControl (replicate 1) | 299 bp |  |
| H2Bub1_siControl (replicate 2) | 274 bp |  |
| Input_siControl (replicate 1) | 280 bp |  |
| Input_siRNF40 (replicate 1) | 267 bp |  |

**Supplementary Materials and Methods**

**Animal handling and mouse model generation**

Animals were housed in the animal facility of the European Neuroscience Institute (ENI) of Göttingen under specific pathogen-free (SFP) conditions. The generation of mice harboring a conditional *Rnf40* allele was previously described by our group (2). All mice had a FVB/N background. Specifically, the *Rnf40*^loxP^ mice were crossed with MMTV-Cre and MMTV-Erbb2 mice to allow co-expression of the Cre-recombinase and of the rat *Erbb2* proto-oncogene in mammary epithelial cells (4,5). Tumors animals were monitored twice a week. Growing tumors were detected by palpation and size was measured with a caliper. The measurements were performed in a blinded way by two independent investigators. Three cohorts of animals were analyzed in this study: n=28 *Rnf40*^wt/wt^, n=12 *Rnf40*^fl/wt^ and n=14 *Rnf40*^fl/fl^ animals. To avoid bias caused by short tumor monitoring time, the number of animals included in “tumor burden” and “tumor growth kinetics” analyses was reduced (n=10 *Rnf40*^fl/wt^; n=13 *Rnf40*^fl/fl^, and n=22 *Rnf40*^wt/wt^; n=9 *Rnf40*^fl/wt^; n=9 *Rnf40*^fl/fl^, respectively).

**Histology of human tissue microarrays (TMAs) and murine tumors**

176 primary mammary tumors and 78 brain metastases samples of known subtype on two TMAs, kindly provided by Prof. Harriet Wikman-Kocher, Prof. Klaus Pantel (Tumor Biology Institute, University of Hamburg-Eppendorf) and Prof. Markus Glatzel (Institute for Neuropathology, University of Hamburg-Eppendorf, Hamburg, Germany), were successfully stained for RNF40 and H2Bub1 using IHC.

Paraffin-embedded tissue microarrays as well murine tumors were deparaffinized in xylol for 20 min and rehydrated with subsequent incubations in 50% isopropanol/50% xylol, 100% isopropanol, 100%, 90% and 70% ethanol, and finally tap water for 5 min each. For hematoxylin and eosin (H&E) staining, nuclei were stained with hematoxylin solution (Carl Roth GmbH) for 1 min. Excess dye was removed using running tap water for 5 min. Counterstaining with eosin (Carl Roth GmbH) was performed for 5-10 min. For immunohistochemical staining (IHC), upon rehydration, antigen retrieval was performed by boiling slides in EDTA buffer (1 mM EDTA, pH 8, 0.1% Tween 20) or citric buffer (10 mM citric acid, pH 6, 0.1% Tween 20) for 10 min in a pressure cooker. After allowing tissue sections to cool slowly, endogenous peroxidase was inactivated with 3% hydrogen superoxide in PBS for 45 min and unspecific antigen binding was blocked with 5% bovine serum albumin (BSA, Merck) and 1% donkey serum (Dianova GmbH) in PBS (blocking solution) for 1 hour at room temperature in a humid chamber. Afterwards, primary antibodies were diluted in blocking solution and sections were incubated overnight in the humid chamber at 4 ^o^C. Sections were next washed twice with 0.1% Tween 20 in PBS (PBS-T) and incubated with biotinylated secondary antibodies blocking solution (1:200 dilution) for 1 hour in the humid chamber at room temperature. After a wash step with PBS-T, Avidin-Peroxidase conjugate (Sigman-Aldrich) diluted in PBS (1:1000) was applied on the sections for 90 min at room temperature in the humid chamber. Finally, staining was developed using 3,3’-diaminobenzidin-tetrahydrochloride (DAB) with 1% hydrogen superoxide in PBS and counterstained using hematoxylin. Slides were washed under running tap water for 5 min and dehydrated in increasing concentrations of ethanol, isopropanol, xylol and mounted with Roti®-Histokitt mounting medium (Carl Roth GmbH). Table S5 and S6 list the antibodies, dilutions and corresponding antigen retrieval buffers used in this study.

For the evaluation of the TMAs staining, two independent blinded observers monitored the staining intensity (0 = negative, 1 = very weak staining, 2 = weak staining, 3 = moderate staining and 4 = strong staining). The score was determined based on the 25% most intensively stained tumor cells. The two evaluations were finally compared and reevaluated if a discrepancy was identified. Because of the relatively low number of HER2^+^-BC, we classified the samples as RNF40^low^ (staining scores 1 and 2) and RNF40^high^ (staining scores 3 and 4) in our analyses. Samples were classified along their intrinsic subtypes based on published procedure (6): luminal A: ER-positive/PR-positive/HER2-score 0, luminal B: ER-positive/PR-positive/HER2-score 1-3, HER2-positive: ER-negative/PR-negative/HER2-score 1-3, TNBC: ER-negative/PR-negative/HER2-score 0.

**Analysis of publically available patient datasets**

Kaplan-Meier Plotter

The Kaplan-Meier Plotter (<http://kmplot.com>) platform was used to study the relapse-free survival (RFS) of HER2-positive patients with low or high expression of *RNF40* (7). Following settings were used here:

*RFS:* Affymetrix probe identity number for *RNF40* gene expression 206845_s_at; auto-select best cutoff mode; HER2-positive BC patients selection criteria: ER-negative, PR-negative, HER2-positive.

*OS:* Affymetrix probe identity number for *RNF40* gene expression 239801_at; auto-select best cutoff mode; HER2-positive BC patients selection criteria: HER2-positive intrinsic subtype.

*DMFS:* Affymetrix probe identity number for *RNF40*, *VAV3*, *ROCK1*, *LIMK2* gene expression are 206845_at, 218806_s_at, 235854_x_at and 202193_at, respectively; auto-select best cutoff mode; HER2-positive BC patients selection criteria: intrinsic subtype

*TCGA*

The TCGA-derived BReast AdenoCarcinoma (BRCA) dataset was retrieved from the Xena browser (<https://xenabrowser.net>) (8) online platform to analyze the impact of *RNF40* expression level on the OS of HER2-positive BC patients (classified along the PAM50). A cutoff of 11.53 normalized reads counts was selected to discriminate low and high *RNF40*-expressing patients. The results were finally plotted with Graphpad Prism v8.01. TCGA normalized expression levels of *RNF40* in normal mammary tissues and breast adenocarcinoma was extracted from the same platform.

*DepMap*

The CERES scores for the genetic essentiality of all screened HER2-positive breast cancer cell lines were performed by Meyers et al. (9) and are publically available at the DepMap portal (https://depmap.org/portal/) under the dataset name ‘’CRISPR (Avana) Public 19Q4’’. CERES score values were plotted as a scatter plot using Graphpad Prism v8.01.

**Cell culture**

HCC1954 and SKBR3 cells were purchased from ATCC company (following Table S1) and cultivated using the recommended medium supplemented with 10% fetal bovine serum (FBS) and 1% penicillin/streptomycin at 37°C and 5% CO_2_.

**siRNA transfections**

Transfections were performed using Lipofectamine® RNAiMAX (Invitrogen) according to the manufacturer’s guidelines. siGENOME SMARTpool siRNA (Dharmacon) are shown in Table S2.

**Plasmid transfections**

Transfections were performed using TrasiT-2020 (Mirus) according to the manufacturer’s instructions. An overexpressing vector harboring CDK9 cDNA in the pSG5 backbone was utilized to overexpress CDK9 in HCC1954 cells. The empty vector was used as a control. Briefly, 240,000 cells per well in a 6-well plate were seeded and the following day the plasmid transfection was performed. At 88 hours post-transfections, cells were harvested for RNA extraction.

**Proliferation, colony and sphere formation assay**

All experiments were performed in biological triplicates.

*HCC1954 cells*:

*Proliferation assay*: 24 hours post-transfection, 1,000 cells were seeded in a 96-well plate. Cell confluency was recorded every 12 hours for 6 days using an IncuCyte® Live Cell Analysis System (Sartorius AG). *Clonogenic and tumor sphere formation assay*: 24 hours post-transfection, 500 (for colony formation assay) and 1000 (for tumor sphere formation assay) cells were seeded in a 6-well and 96-well low adherent plate, respectively. 15 days after seeding, colonies were washed with PBS, fixed with methanol for 10 min and stained with 1% crystal violet in 20% ethanol for 20 min. After a final wash in water, stained colonies were scanned using EPSON perfection V700 PHOTO scanner. 20 days after seeding, spheres were scanned using a Celigo® S imaging cytometer (Nexcelom Bioscience LLC) and quantified. Number of colonies or spheres was assessed using ImageJ.

*SKBR3 cells*:

*Proliferation and clonogenic assay*: 220,000 cells were reverse transfected in biological triplicates in a 6-well plate and re-transfected 96 hours after the first transfection. 24 hours later, 1,000 cells (for proliferation assay) and 500 cells (for colony formation assay) were seeded on a 96-well (adherent) and a 6-well plate, respectively. Proliferating cells were scanned every 2 days using a Celigo® S imaging cytometer (Nexcelom Bioscience LLC). 15 days after seeding, colonies were washed, fixed, stained, scanned and analysed as previously described.

*BT474 cells:*

*Tumor sphere formation assay:* 220,000 cells were reverse transfected in biological triplicates in a 6-well plate and re-transfected 96 hours after the first transfection. 24 hours later, 500 cells per well were seeded in a low adherent 96-well plate. and the culture medium was supplemented with B27 supplements (Gibco, cat.11530536) based on manufacturer’s instructions. 9 days after seeding, tumor spheres were scanned using a Celigo® S imaging cytometer (Nexcelom Bioscience LLC) and quantified.

*ROCK and FAK inhibition*: 30,000 and 220,000 HCC1954 cells were seeded in 12-well (proliferation assay) and 6-well plates (protein extraction), respectively. The following day, medium was replaced with fresh one including 16 μΜ RKI-1447 (CAS 1342278-01-6, Toronto Research Chemicals) or 20 μΜ PF-573228 (14924, Cayman Chemical). Cells for protein isolation were harvested after 12 hours of treatment while cells for proliferation assay were grown for 5 days. Finally, plates were fixed, stained, scanned and analysed as previously described.

*WDR5 inhibition*: 50,000 cells per well in a 12-well plate were seeded. The following day, HCC1954 cells were treated with DMSO or 5 μΜ of the WDR5-specific inhibitor OICR-9429 (Selleckhem) for 5 days to subsequently perform RNA extraction.

*Rescue experiments*: upon RNF40 silencing, 30,000 and 220,000 HCC1954 cells were reverse transfected in biological triplicates in a 12-well (proliferation assay) and 6-well plate (protein isolation), respectively. 24 hours after transfection, medium was replaced with fresh one including 10 μΜ CYM-5441 (S1PR_3_ agonist, SML0680, Sigma-Aldrich) or 10 μΜ lysophosphatidic acid (LPA, CAS 325465-93-38, Santa Cruz). For protein isolation, cells were protein harvested 72 hours post-transfection while cells for proliferation assay were grown for 5 days.

Co-treatment of S1PR_3_ agonist- or LPA-induced with ROCK inhibition: 30,000 HCC1954 cells were seeded in a 12-well plate. The following day, medium was replaced with fresh one including 10 μΜ CYM-5441 or 10 μΜ LPA plus 16 μΜ RKI-1447 and cells were grown for 5 days. Finally, plates were fixed, stained, scanned and analysed as previously described.

**Annexin V and caspase 3/7 activity assay**

*Annexin V assay (modified from* (10)*):* 72 hours post-transfection, cells were washed with PBS and resuspended in 1x Binding buffer (10 mM HEPES, 0.14 M NaCl, 2.5 m M CaCl_2_ pH: 7.4) at a concentration of 10^6^ cells/ml. 100 μl of the cell suspensions were transferred to sterile tubes, 5 μl of Annexin V-FITC (Southern Biotech) and 1 µl of propidium iodide (1mg/ml, Sigma Aldrich) was added in each sample and suspension was incubated for 15 min at room temperature in the dark. Finally, 400 μl of 1x Binding buffer was added to each tube and samples were analysed using a Guava EasyCyte Plus flow cytometer from Guava Technologies.

*Caspase 3/7 activity assay*: 24 hours post-transfection, 1,000 cells were seeded on a 96-well plate containing in each well 100 μl medium containing caspase 3/7 fluorescent substrate (CS1-V0002-1, ViaStain^TM^ Live Caspase 3/7 Detection Kit) and Hoechst 33342, according to the manufacturer’s instructions (CS1-V0002(3)-1, ViaStain^TM^ Live Caspase 3/7 Detection Kit) and supplemented with either 10 μΜ CYM-5441 or vehicle. Scanning was carried out with a Celigo® S imaging cytometer (Nexcelom Bioscience LLC). Output data were normalized to the first day of scanning for each condition.

**Migration assays**

*Trans-well migration assay*: 120,000 HCC1954 cells were reverse transfected in biological triplicates, and re-transfected 96 hours later. The following day, cells were serum starved for 8 hours and then collected, counted using trypan blue and 50,000 viable cells were seeded on each trans-well inserts (Corning, 24-well insert, 8 μm pore) while normally supplemented medium was added beneath the inserts. 48 hours after seeding, the inserts were washed with PBS and cells that have not migrated through the membrane were removed with a cotton Q-tip. Migrated cells were then fixed with methanol for 10 min, stained with crystal violet, scanned and analysed as previously described (see method for proliferation assay).

*Gap-closure assay*: 120,000 HCC1954 cells were reverse transfected in biological triplicates, and re-transfected 96 hours later. Cells were collected, counted using trypan blue and 70,000 viable cells were seeded on each silicone insert (ibidi, 2-well μ-Dish 35 mm). Once cells became adherent, silicone chambers were removed and photos were taken using a Nikon Eclipse TS100 inverted microscope at time point 0 and 23 hours after silicone insert removal. Gap closure was measured using ImageJ and normalized to time point 0 hours.

**Immunofluorescence microscopy**

10-15,000 cells were reverse transfected on coverslips in biological triplicates in a 6-well plate as previously described. 72 hours post-transfection cells were washed with PBS and fixed with 4% paraformaldehyde in PBS for 20 min. Thereafter, cells were permeabilized with 1% Triton X-100 in TBS for 10 min, washed once with TBS and blocked with blocking solution (3% BSA in TBS-T) for 1 hour in a humid chamber. The primary antibody was diluted in blocking solution and applied on the coverslips for overnight incubation in a dark humid chamber at 4°C. The following day, coverslips were washed three times with TBS-T and incubated with fluorophore-conjugated secondary antibodies and DAPI (1:1000 dilution) dissolved in blocking solution, for 1 hour in a dark humid chamber. For F-actin staining, Alexa555 conjugated to phalloidin (Abnova) was added to the secondary antibody solution at 1:400 dilution. Coverslips were washed three times with TBS-T. Finally, coverslips were mounted on microscope slides. Pictures were taken with a Zeiss LSM 510 Meta confocal microscope. Fluorescence intensity was quantified using ImageJ.

Fluorescence intensity quantification: photographed areas were processed in ImageJ. To quantify Ki67 (*in vitro*) as well p-cofilin (*in vivo*) staining, the DAPI channel was utilized as reference to determine cell nuclei regions. Finally, Ki67 or p-cofilin staining intensity was measured for every nucleus. To quantify F-actin staining intensity, the area covered by cells was determined based on the phalloidin channel. The average F-Actin staining intensity was determined in this area for each picture.

To quantify the focal adhesion area and their staining intensity, a particle analysis was performed on the vinculin channel. Used antibodies are listed at Tables S5 and S6.

**Protein isolation and western blot analysis**

Protein isolation: Radioimmunoprecipitation Assay Buffer (RIPA; 10 mM Tris-Cl pH 8, 1 mM EDTA, 1% v/v Triton X-100, 0.1% sodium deoxycholate, 0.1% SDS, 140 mM NaCl) supplemented with protease and phosphatase inhibitors was used (1 μM activated orthovanadate, 10 mM β-glycerophosphate disodium salt hydrate, 10 mM Pefablock, 10 mM N-Ethylmaleimide, 1 mM Aprotinin/Leupeptinin, 1mμM NaF, 1 μM iodoacetic acid). Cells were washed once with PBS and 200 μl of RIPA buffer was added to each well (6 well plate). After 10 min incubation on ice, cells were scraped and lysates were sonicated for three cycles 30s on/off each using a Bioruptor (Diagenode). Laemmli buffer (375 mM Tris/HCl, 10% SDS, 30% glycerol, 0.02% bromophenol blue, 9.3% DTT) was added to each lysate and cooked at 95°C for 5 min before protein separation with a 10 to 12% polyacrylamide gel. Proteins were transferred to nitrocellulose membrane (0.45 µm pore, Immobilon, Millipore), blocked with 5% skimmed milk in TBS-T for 1 hour and incubated with primary antibody overnight at 4°C. The day after, membrane was washed with TBS-T, incubated 1 hour with secondary antibody at room temperature. After a final wash step, protein detection was achieved with the Millipore substrate in a BioRad ChemiDoc^TM^ imager. Used primary antibodies are listed at Table S5 and S6.

**RNA isolation and quantitative RT-PCR (qRT-PCR)**

RNA isolation and quantitative RT-PCR were performed as previously described (11,12). Briefly, 72 hours post-transfection, cells were washed wit PBS and lysed in 500 µl Qiazol (Qiagen). For RNA extraction from tissues, 50-100 mg frozen tissue was homogenized with 0.5-1 ml Qiazol with three cycles of 10-15 sec/2,000 rpm in a PowerLyzer24 (MoBio Laboratories). Lysates were then collected and RNA was extracted, as previously described (11,12). Reverse transcription of 1 µg RNA was performed using M-MuLV reverse transcriptase (NEB) with random primers according to the manufacturer’s instructions. Expression of specific genes was finally estimated by quantitative real-time PCR using a CFX Connect™ Real-Time System (Bio-Rad). Gene expression levels were normalized relative to the *RPLP0* house keeping gene. qRT-PCR program: 1x 2 min-95 ^o^C, 40x 10 sec-95 ^o^C followed by 1x 30 sec-60°C. Primers (Table S3) were designed using the design tool (http://biotools.umassmed.edu/bioapps/primer3_www.cgi) and were ordered from Sigma-Aldrich (Germany).

**Chromatin Immunoprecipitation (ChIP)**

72 hours post-transfection, cells were crosslinked with 1% formaldehyde for 20 minutes and quenched by glycine (125mM final concentration) at room temperature for 5 min. Subsequently, fixed cells were scraped and nuclear pellets were collected and washed with Nelson buffer (150 mM NaCl, 20 mM EDTA, 50 mM Tris-HCl (pH 7.5), 0.5% v/v NP-40, 1% v/v Triton-X-100, 20 mM NaF). Nuclei were then sonicated in Gomes lysis buffer (150mM NaCl, 20 mM EDTA, 50 mM Tris-HCl (pH: 8), 1% v/v NP-40, 0.5% v/v sodium deoxycholate, 20 mM NaF, 0.1% SDS) for 20 cycles using a Bioruptor Pico (Diagenode) with each cycle setup of 30 s on/off. After performing shearing check, samples were precleared by incubation with 50% slurry of sepharose beads (GE Healthcare), centrifuged and supernatants were incubated with H2Bub1, H3K4me3 antibody or control rabbit IgG (1μg, C15410206, Diagenode) overnight. Protein A-sepharose beads were added to samples and incubated for 2 hours at 4°C, then washed [1 time with Gomes lysis buffer, 2 times with Gomes wash buffer (0.5 M LiCl, 0.02 M EDTA, 0.1 M Tris-EDTA, 1% NP-40, 0.02 M NaF, 1% sodium deoxycholate) and two times again with Gomes lysis buffer]. DNA-beads complexes were washed once more with 1 mM Tris and 10 mM EDTA buffer (TE) before DNA was extracted. Samples were run in three biological replicates per condition. For ChIP antibodies, please refer to Table S5. H2Bub1 and H3K4me3 ChIP efficiency was validated via ChIP-qPCR for genomic regions known to be H2Bub1 positive (body region of *GAPDH*) or H3K4me3 positive (TSS-proximal region of *HNRNPK*). The signal was normalized to input DNA and presented as percent input for triplicates in each condition. The validation of H3K4me3 regulated regions was performed by ChIP-qPCR following the same procedure. Primer list available in Table S4.

**Library preparation for RNA and ChIP-seq and next generation sequencing**

RNA sequencing libraries were generated with the NEXTflex^TM^ Rapid Illumina Directional Kit (Biooscientific, Catalog #NOVA-5138-07) according to the manufacturer’s instructions. RNA library underwent amplification in a thermal cycler using the following program: 1 cycle of of 37^o^C-30 min, 98 ^o^C-2 min and 15 cycles of [98 ^o^C-30 sec, 1x 65 ^o^C-30 sec, 1x 72 ^o^C-60 sec] and one cycle of 72 ^o^C-4 min.

ChIP libraries were generated with the Microplex Library Preparation kit v2 (Diagenode, C05010011) and were amplified using the following program: 1 cycle of [72 ^o^C-3 min, 85 ^o^C-2 min, 1x 98 ^o^C-2 min], 4 cycles of [98 ^o^C-20 sec, 67 ^o^C-20 sec, 72 ^o^C-40 sec] and 16 (H3K4me3, input) or 17 cycles (H2Bub1) of [98 ^o^C-20 sec and 72 ^o^C-50 sec].

The quality and size of the libraries was examined using the high sensitivity DNA kit (Agilent) on the Agilent Bioanalyzer 2100 (Table S7). Finally, the concentration of the mRNA- and ChIP libraries was estimated with a Qbit (Invitrogen), multiplexed in 2 nM pooled libraries and sequenced (single-end, 50 bp) on a HiSeq4000 (Illumina) in the Transcriptome and Genome Analysis Laboratory (TAL) at the University Medical Center of Göttingen.

**Bioinformatic analysis of mRNA-sequencing data**

Fastq files were uploaded and processed in the Galaxy environment (<https://galaxy.gwdg.de>). Qualitative of the sequencing data was assessed using FastQC (version 0.72)(13). Fastq files were trimmed for the first 11 bp using the FASTQ Trimmer tool (version 1.0.0) (14). Output data were aligned to the human reference genome hg19 (downloaded from www.ensembl.org) using the TopHat Gapped-read mapper (version 2.1.1)(15). Aligned reads were then assigned to the respective genomic features using featureCounts (version 1.4.6.p5) and, finally, DESeq2 (version 2.11.39) was used to identify significantly differentially regulated genes (16).

Matrix visualisation of actin cytoskeleton gene signatures in Fig.3F and 5F were created using the Morpheus tool (<https://software.broadinstitute.org/morpheus/>). Gene Set Enrichment Analyses (GSEA) were performed with normalized counts of siControl and siRNF40 conditions using following specific settings: 1,000 permutations, type: gene set and a maximum size of sets of 1,000) (17). Pathway enrichment analysis for all RNF40-dependent genes was performed using the online tool Enrichr (18).

mRNA-seq data (in FPKM) from HER2+ BC patients were retrieved from the Genomic Data Commons Data Portal (GDC). FPKM count tables were sorted along RNF40 expression (*RNF40*^low^=27, *RNF40*^high^=29) in a common count table and subsequently subjected to GSEA analysis. Volcano plot of normalized enrichment score and FDR of all gene ontology signatures of biological processes (GO_BP) were plotted using Graphpad Prism v8.01.

**Bioinformatic analysis of ChIP-sequencing data**

Fastq files were uploaded and processed in the Galaxy environment (<https://galaxy.gwdg.de>). Qualitative of the sequencing data was assessed using FastQC (version 0.72) (13). Respective reads were aligned to the human reference genome hg19 using Bowtie2 (version 2.3.2.2) (19) using the “very sensitive end-to-end” mapping mode. Output bam files were filtered using the “Filter SAM or BAM” tool (version 1.1.2) (20) using a minimum MAPQ quality score of 5. Subsequently, PCR duplicates were removed using the “RmDup” tool (version 2.0.1) (20). BigWig files of the H3K4me3 and H2Bub1 tracks were created using the ”bamCoverage” (version 2.5.1.1.0 and version 3.0.1.0, respectively, (21)) (normalisation to reads per kilobase per million; RPKM) from Deeptools in the Galaxy environment. Reads in this step were extended using the average fragment size of each sample calculated from the library quality screening step (Table S7). A minimum mapping quality was set at 5 while a black list chromatin region hg19-based file, downloaded from <https://sites.google.com/site/anshulkundaje/projects/blacklists>, was used to exclude regions with anomalous signal. For peak calling of H2Bub1 and H3K4me3 data, “MACS2 callpeak” (version 2.1.1.20160309.5, (22)) was used with the respective input files as background (settings: FDR<0.05, broad regions, cut-off for broad regions at 0.05). Finally, to generate heatmaps and aggregate plot profiles from the given regions, “computeMatrix”, “plotHeatmap” and “plotProfile” (version 3.2.0.0.0) from the DeepTools suite were used, respectively.

To identify and call more precisely H3K4me3-regions regulated upon RNF40 silencing, we used the “bigwigCompare” (version 3.2.0.0.0) from DeepTools of the Galaxy environment to compute the fold change values between siRNF40 and siControl conditions (bin size = 50 bp). Bins with read density over 5 and │fold change│≥0.8 (regulated regions) or │fold change│<0.8 (for unregulated) were kept and merged using the “MergeBed” (version 2.27.1) from DeepTools with a maximum distance of 250 bp. Subsequently, the output region file underwent differential binding analysis as well principal component analysis using R-studio Bioconductor R package (Diffbind, (23)) run on R version 3.3.1 according to the instructions. We limited our analyses to promoter associated regions and assigned significantly differentially regulated H3K4me3 regions to the respective genes. Visualisation of those regions was performed using a volcano plot script from the bioconductor website (<https://www.bioconductor.org>) run on R version 3.3.1.

For the aggregate profile of H3K4me3 peak width changes in Figures S5C, 5F and S5F, the peak width was calculated using the following equation and expressed in log2scale:

siControl (peak end-peak start)

Peak width (bp)=

siRNF40 (peak end-peak start)

For the region-gene association of all “Diffbind”-defined regions with loss of H3K4me3 (Fig.S5B), the online Genomic Region Enrichment Analysis Tool (GREAT, (24)) was used with human reference genome hg19 and 1kb upstream and 5kb downstream from the nearest TSS as presets. For visualisation of the BigWig files across the human reference genome, Integrative Genomics Viewer tool (version 2.4.8, (25)) was used. For performing pathway enrichment analysis for genomic regions with loss of H3K4me3 upon RNF40 silencing, the online tool Enrichr was used (18).

**Supplementary References**

1. Karpiuk, O. *et al.* The Histone H2B Monoubiquitination Regulatory Pathway Is Required for Differentiation of Multipotent Stem Cells. *Mol. Cell* **46,** 705–713 (2012).

2. Xie, W. *et al.* RNF40 regulates gene expression in an epigenetic context-dependent manner. *Genome Biol.* **18,** 32 (2017).

3. Najafova, Z. *et al.* RNF40 exerts stage-dependent functions in differentiating osteoblasts and is essential for bone cell crosstalk. *Cell Death Differ.* 1–15 (2020). doi:10.1038/s41418-020-00614-w

4. Shema, E. *et al.* The histone H2B-specific ubiquitin ligase RNF20/hBRE1 acts as a putative tumor suppressor through selective regulation of gene expression. *Genes Dev.* **22,** 2664–2676 (2008).

5. Guy, C. T. *et al.* Expression of the neu protooncogene in the mammary epithelium of transgenic mice induces metastatic disease. *Proc. Natl. Acad. Sci. U. S. A.* **89,** 10578–82 (1992).

6. Wagner, K. U. *et al.* Cre-mediated gene deletion in the mammary gland. *Nucleic Acids Res.* **25,** 4323–4330 (1997).

7. Tang, P. & Tse, G. M. Immunohistochemical Surrogates for Molecular Classification of Breast Carcinoma: A 2015 Update. *Arch. Pathol. Lab. Med.* **140,** 806–14 (2016).

8. Györffy, B. *et al.* An online survival analysis tool to rapidly assess the effect of 22,277 genes on breast cancer prognosis using microarray data of 1,809 patients. *Breast Cancer Res. Treat.* **123,** 725–31 (2010).

9. Goldman, M. *et al.* The UCSC Xena Platform for cancer genomics data visualization and interpretation. Preprint at https://www.biorxiv.org/content/10.1101/326470v6

10. Meyers, R. M. *et al.* Computational correction of copy number effect improves specificity of CRISPR-Cas9 essentiality screens in cancer cells. *Nat. Genet.* **49,** 1779–1784 (2017).

11. Lakshmanan, I. & Batra, S. Protocol for Apoptosis Assay by Flow Cytometry Using Annexin V Staining Method. *BIO-PROTOCOL* **3,** (2013).

12. Prenzel, T. *et al.* Estrogen-dependent gene transcription in human breast cancer cells relies upon proteasome-dependent monoubiquitination of histone H2B. *Cancer Res.* **71,** 5739–5753 (2011).

13. Mishra, V. K. *et al.* Histone deacetylase class-I inhibition promotes epithelial gene expression in pancreatic cancer cells in a BRD4- and MYC-dependent manner. *Nucleic Acids Res.* **45,** 6334–6349 (2017).

14. Blankenberg, D. *et al.* Manipulation of FASTQ data with Galaxy. *Bioinformatics* **26,** 1783–1785 (2010).

15. Conway, E., Healy, E. & Bracken, A. P. PRC2 mediated H3K27 methylations in cellular identity and cancer. *Curr. Opin. Cell Biol.* **37,** 42–48 (2015).

16. Trapnell, C., Pachter, L. & Salzberg, S. L. TopHat: Discovering splice junctions with RNA-Seq. *Bioinformatics* **25,** 1105–1111 (2009).

17. Love, M. I., Huber, W. & Anders, S. Moderated estimation of fold change and dispersion for RNA-seq data with DESeq2. *Genome Biol.* **15,** (2014).

18. Subramanian, A. *et al.* Gene set enrichment analysis: A knowledge-based approach for interpreting genome-wide expression profiles. *Proc. Natl. Acad. Sci. U. S. A.* **102,** 15545–15550 (2005).

19. Kuleshov, M. V. *et al.* Enrichr: a comprehensive gene set enrichment analysis web server 2016 update. *Nucleic Acids Res.* **44,** W90–W97 (2016).

20. Langmead, B. & Salzberg, S. L. Fast gapped-read alignment with Bowtie 2. *Nat. Methods* **9,** 357–359 (2012).

21. Li, H. *et al.* The Sequence Alignment/Map format and SAMtools. *Bioinformatics* **25,** 2078–2079 (2009).

22. Ramírez, F. *et al.* deepTools2: a next generation web server for deep-sequencing data analysis. *Nucleic Acids Res.* **44,** W160-5 (2016).

23. Feng, J., Liu, T., Qin, B., Zhang, Y. & Liu, X. S. Identifying ChIP-seq enrichment using MACS. *Nat. Protoc.* **7,** 1728–1740 (2012).

24. Ross-Innes, C. S. *et al.* Differential oestrogen receptor binding is associated with clinical outcome in breast cancer. *Nature* **481,** 389–393 (2012).

25. McLean, C. Y. *et al.* GREAT improves functional interpretation of cis-regulatory regions. *Nat. Biotechnol.* **28,** 495–501 (2010).

26. Robinson, J. T. *et al.* Integrative genomics viewer. *Nat. Biotechnol.* **29,** 24–26 (2011).

**Supplementary Figure Legends**

**Fig.S1 RNF40 and H2Bub1 loss are rare events and do not advantage tumor growth in HER2-positive malignancies of the breast. A:** Representative pictures of low and high H2Bub1 and RNF40 staining in HER2-positive brain metastases TMA specimens. **B:** 2x2 contingency table of RNF40^low^- and RNF40^high^-expressing primary tumors and brain metastases biopsies. Fisher exact test. **C:** Overall survival (OS) and Distant Metastasis-Free Survival (DMFS) analysis of *RNF40*^low^- and *RNF40*^high^-expressing HER2-positive BC patients, retrieved from TCGA (<https://xenabrowser.net/>) and KM-plotter (kmplot.com), respectively. **D:** 2x2 contingency table of RNF40^low^-, RNF40^high^-, H2Bub1^low^- and H2Bub1^high^-expressing primary tumors compared to their respective tumor grade. Fisher exact test. **E:** *RNF40* expression is in overall higher in breast tumor tissues (n=6470) and in the HER2-enriched BC subtype (n=82), than in their normal healthy counterparts (n=101) (log_2_normalized counts, source=TCGA BRCA dataset). One-way ANOVA (Kruskal-Wallis-test). **F:** Representative pictures of RNF40 and H2Bub1 immunohistochemical staining on *Rnf40*^wt/wt^ normal mammary epithelial tissues adjacent to growing tumors. Scale bar: 100 μm. **G:** Representative pictures of hematoxyline-and-eosin (H&E) stained *Rnf40*^wt/wt^ and *Rnf40*^fl/fl^ tumors. Scale bar: 100 μm. **H:** RT-qPCR validation of the *Rnf40* knockout efficiency in murine primary tumor tissues at the gene expression level. **I:** Quantification of Ki67 positive cells in IHC stained paraffin sections of *Rnf40*^wt/wt^ and *Rnf40*^fl/fl^ tumors (n=7 tumors per mouse cohort, n=2 analyzed pictures per tumor). **p-val<0.01, ***p-val<0.005. Student t-test. **H** and **I**: error bars= SEM.

**Fig.S2: RNF40 loss impairs oncogenic properties of HER2-positive BC cells *in vitro.* A:** Validation of the RNF40 silencing in HCC1954 and SKBR3 cells using qRT-PCR. **B:** Validation of the RNF40 silencing and H2Bub1 decrease in siRNF40-treated SKBR3 cells by western blot analysis. **C:** Tumor sphere formation assay of siControl- and siRNF40-traeted BT474 cells. Student t-test. **D:** Essentiality of *RNF40* in various breast cancer cell lines, as assessed in the CRISPR-Cas9 screen Avana 19Q4 (<https://depmap.org>) **E:** Representative pictures (left panel) and quantification (right panel) of a gap closure assay from siControl- and siRNF40-transfected HCC1954 cells. Error bars: SEM. Student t-test. **p-val<0.01, ***p-val<0.005. White scale bars: 500 μm. All experiments were performed in biological triplicates. Error bars: SEM.

**Fig.S3: RNF40 loss increases apoptosis and impairs the expression of important key-effectors of the actin regulatory pathway in HER2-positive BC cells.** **A:** Western blot analysis of ERK1/2, pERK1/2, AKT, pAKT levels in siControl- and siRNF40-treated SKBR3 cells. **B-C:** List of gene signatures enriched (FDR < 0.25) in siRNF40-treated HCC1954 **(B)** as well as in *RNF40*^low^-expressing HER2+-BC biopsies **(C)** (normalized sequencing data were retrieved from TCGA portal; GSEA analysis, HALLMARK gene sets). **D:** Western blot analysis of cl-PARP (cleaved PARP) and fl-PARP (full-length PARP) levels in siControl- and siRNF40-treated SKBR3 cells. **E:** List of enriched gene signatures in HCC1954 cells upon siControl-treatment (Enrichr analysis, KEGG 2019 database). **F:** Validation of the *VAV3*, *ROCK1*, *LIMK2* and *PFN2* downregulation in siRNF40-treated SKBR3 cells via qRT-PCR. **G:** Western blot analysis of ROCK1 and VAV3 protein levels in siControl- and siRNF40-treated SKBR3 cells. All qRT-PCRs and western blot analyses were performed in biological triplicates. Student t-test. *p-val<0.05. Error bars: SEM.

**Fig.S4: RNF40 controls the actin regulatory pathway to sustain the viability of HER2-positive BC cells *in vitro* and *in vivo*. A:** Representative pictures of immunofluorescence staining of F-actin (phalloidin) in siControl- and siRNF40-treated SKBR3 cells (left panel). Scale bars (red) = 50 μm. Quantification of F-actin intensity in the respective conditions (right panel). Mann-Whitney test. **B:** Western blot analysis of RNF40 levels in siControl-, siRNF40- and RKI-1447-treated HCC1954 cells (16 μΜ, ROCK inhibitor). **C-D:** Crystal violet staining of HCC1954 cells showing a loss of confluency upon RKI-1447 (16 µM, ROCK inhibitor) **(C)** or PF-373228 (20 µM, FAK inhibitor) treatment **(D)** compared to DMSO control. **E:** Cleaved caspase 3 and 7 activity kinetics measurement in siControl- and siRNF40 treated HCC1954 cells with and without S1PR_3_ agonist treatment. **F:** Crystal violet staining of siControl- and siRNF40-treated HCC1954 cells with and without rescue through 10 μM LPA treatment. Quantification of cell confluency (right panel). Student t-test. **G-H:** Crystal violet staining of vehicle and RKI-1447 (16 µM) treated HCC1954 cells with and without 10 µM S1PR_3_ **(G)** agonist or 10 µM LPA **(H)** treatment. **I-J:** GSEA profile of the ‘’GO_ACTIN_FILAMENT_BUNDLE_ORGANISATION’’ gene set enriched in *RNF40*^high^-expressing HER2^+^-BC biopsies **(I)**. Table of all significantly enriched (FDR<0.25) gene sets associated with H2Bub1, actin dynamics, focal adhesion and apoptosis **(J)**. Data were retrieved from TCGA (<https://portal.gdc.cancer.gov/>). **E** and **F**: Student t-test *p-val<0.05 , **p-val<0.01, ***p-val<0.005. All experiments were performed in biological triplicates. Error bars: SEM.

**Fig.S5: RNF40 regulates gene expression of important members of the RHO-ROCK axis in a H2Bub1-H3K4me3 dependent manner.** **A:** Immunofluorescence staining of H2Bub1 in DMSO- and CDK9i-treated HCC1954 cells (BAY-1251152, 120 nΜ, 48 hours). Scale bar: 100 μm. **B:** Crystal violet staining of HCC1954 cells treated with DMSO- or CDK9i (BAY-1251152, 120 nΜ, 5 days). **C:** Immunofluorescence staining of F-actin and RNF40 in DMSO- and CDK9i-treated HCC1954 cells (BAY-1251152, 120nΜ, 48 hours). Scale bar: 100 μm. **D:** qRT-PCR of the actin regulatory genes in empty vector- and CDK9-overexpressing HCC1954 cells. Student t-test. **E:** Principal component analysis from Differential Binding Analysis results on H3K4me3 occupied regions in siControl- and siRNF40-treated HCC1954 cells. **F:** “GREAT” cis-regulatory region analysis showing the genomic distribution of H3K4me3 loss regions upon RNF40 silencing. **G:** H3K4me3 peak width change upon RNF40 silencing of regulated and unchanged regions identified with a DiffBind analysis. One-way ANOVA (Kruskal-Wallis-test). **H:** Pathway enrichment analysis with the web-based Enrichr tool for all genomic regions loosing H3K4me3. **I:** ChIP-qRT-PCR validating the loss of H3K4me3 occupancy at the 3’-prime end of *ROCK1*, *LIMK2* and *VAV3* peaks (right panel). Integrative genomic viewer (IGV) tracks of H3K4me3 in siControl- and siRNF40-treated HCC1954 cells. Dotted lines represent the used ChIP-qRT-PCR primers to confirm the H3K4me3 narrowing (left panel). All qRT-PCRs were performed in biological triplicates. Student t-test. Error bars: SEM. *p-val<0.05.***p-val<0.005.

**Fig.S6:** **RNF40 regulates gene expression of important members of the RHO-ROCK axis in a H2Bub1-H3K4me3 dependent manner. A:** Violin plot showing the median of H3K4me3 peak width in basal condition at the TSS of Groups A, B and C genes. One-way Anova (Kruskal-Wallis test). **B:** qRT-PCR of some representative Group C genes in DMSO- or CDK9i-treated (BAY-1251152, 120 nΜ, 6 hours) HCC1954 cells. Student t-test.. **C:** Integrative Genomic Viewer (IGV) tracks of H2Bub1, H3K27ac, H3K79me2, H3K36me3 and H3K9ac at Group A, Group B and Group C genes. **D:** Violin plot showing the median of the H3K3me3 peak width change of Group A, B and C genes. One-way Anova (Kruskal-Wallis test). **E:** qRT-PCR of *RRAS2*, *VAV3*, *ROCK1*, *LIMK2* and *PFN2* in DMSO- and OICR9429-treated (WDR5-specific inhibitor) HCC1954 cells (5 μΜ, 5 days). Student t-test. **F:** qRT-PCR of actin regulatory genes in siControl-, siRNF40-, siNELFE, siRNF40+siNELFE-treated HCC1954 cells. Student t-test. **G:** Aggregate plots of H3K79me2, H3K36me3, H3K27ac, H3K9ac or RNApol II occupancy at TSS of group A, B and C* genes in control HCC1954 cells (Accession number: GSE85158, GSE72956). Error bars: SEM: ns=not significant, * p-val<0.05, *** p-val<0.005. All qRT-PCRs were performed in biological triplicates.
