# Supplementary figures and images for: The histone H2B ubiquitin ligase RNF40 is required for HER2-driven mammary tumorigenesis
Source: Cell Death Dis. 2020 Oct 17;11(10):873. doi: 10.1038/s41419-020-03081-w (PMC7568723; doi:10.1038/s41419-020-03081-w)

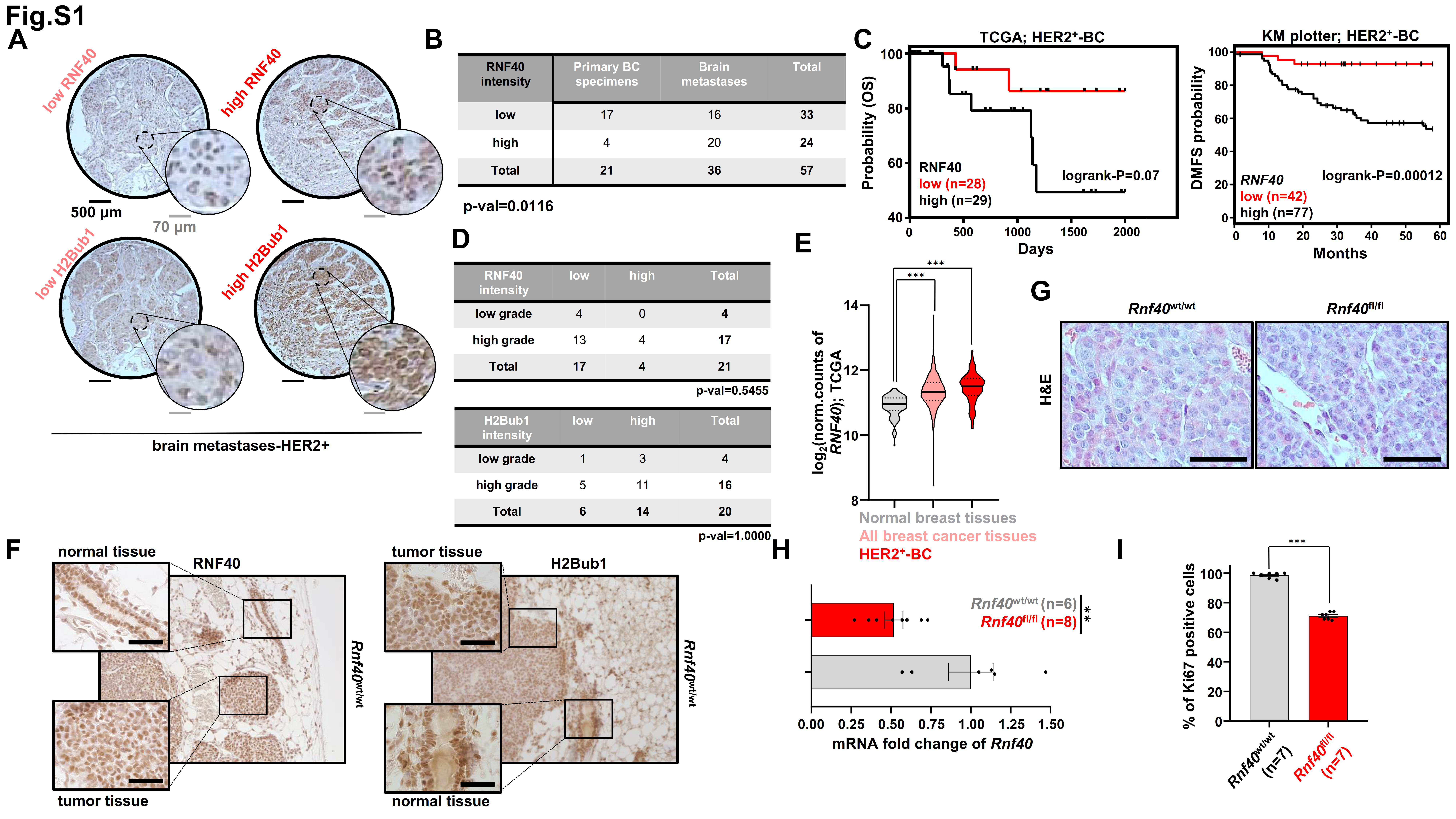

Supplement: Supplementary file 2 — Supplementary Figure S1 [file 41419_2020_3081_MOESM2_ESM.tif]

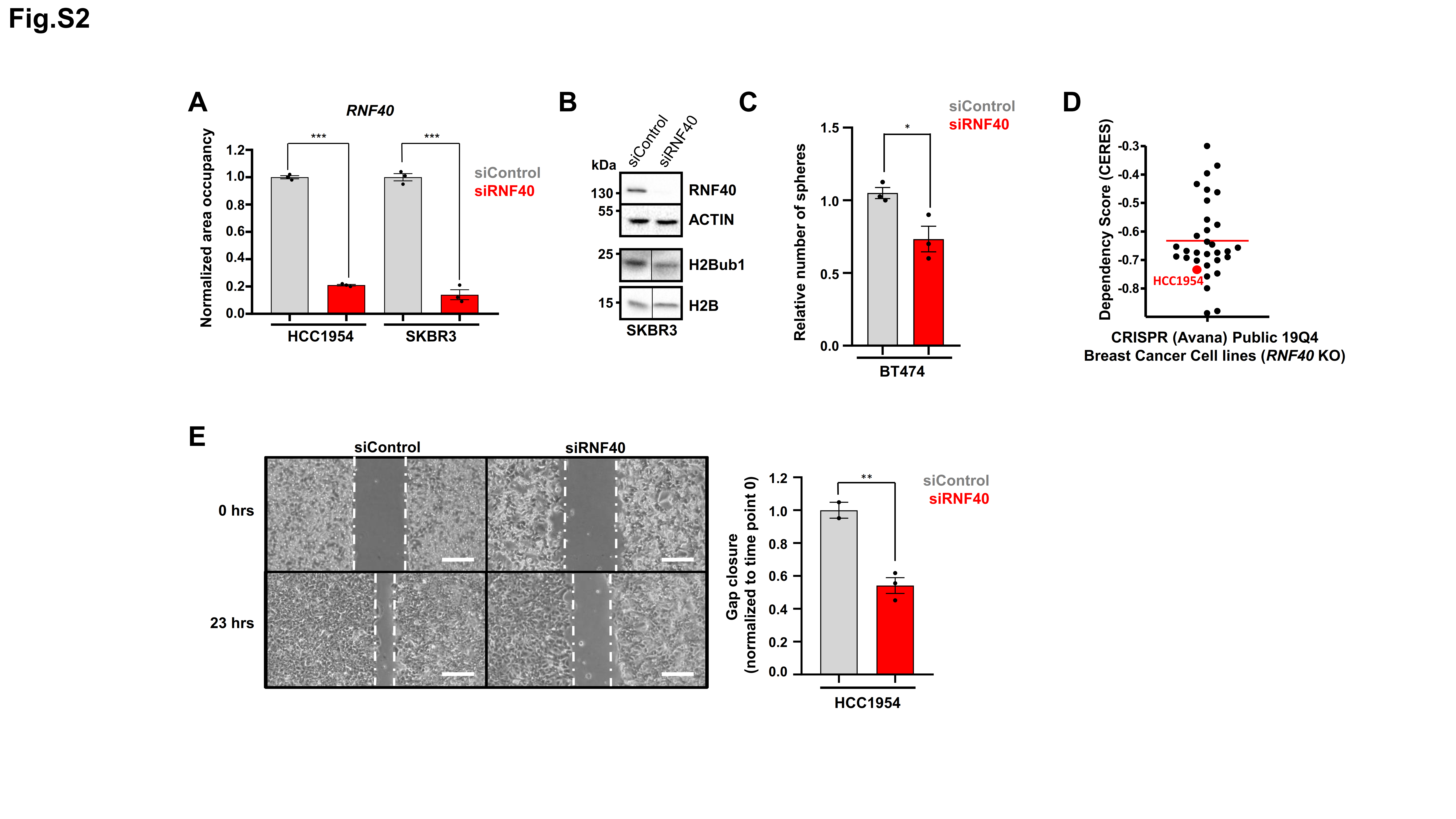

Supplement: Supplementary file 3 — Supplementary Figure S2 [file 41419_2020_3081_MOESM3_ESM.tif]

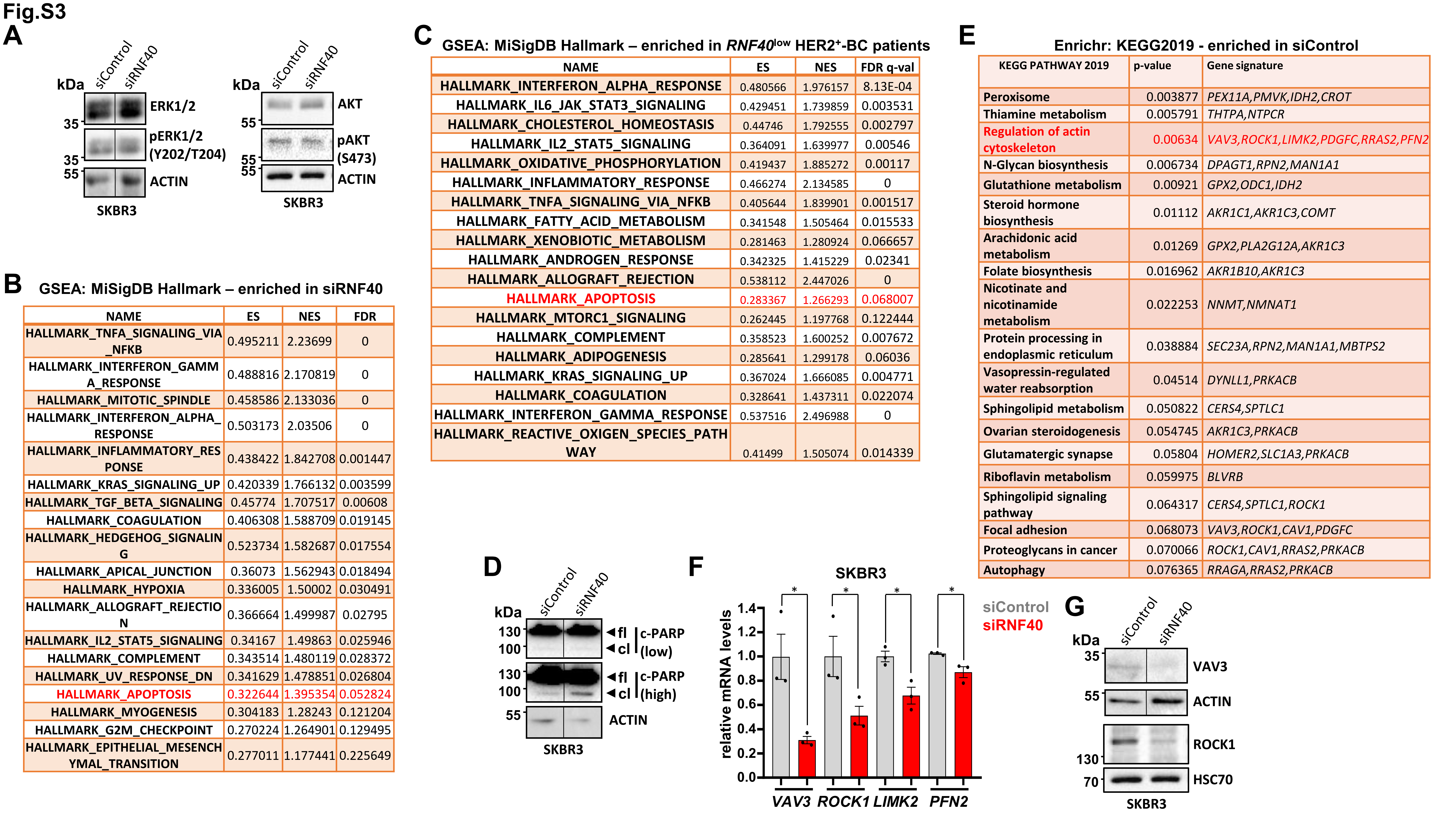

Supplement: Supplementary file 4 — Supplementary Figure S3 [file 41419_2020_3081_MOESM4_ESM.tif]

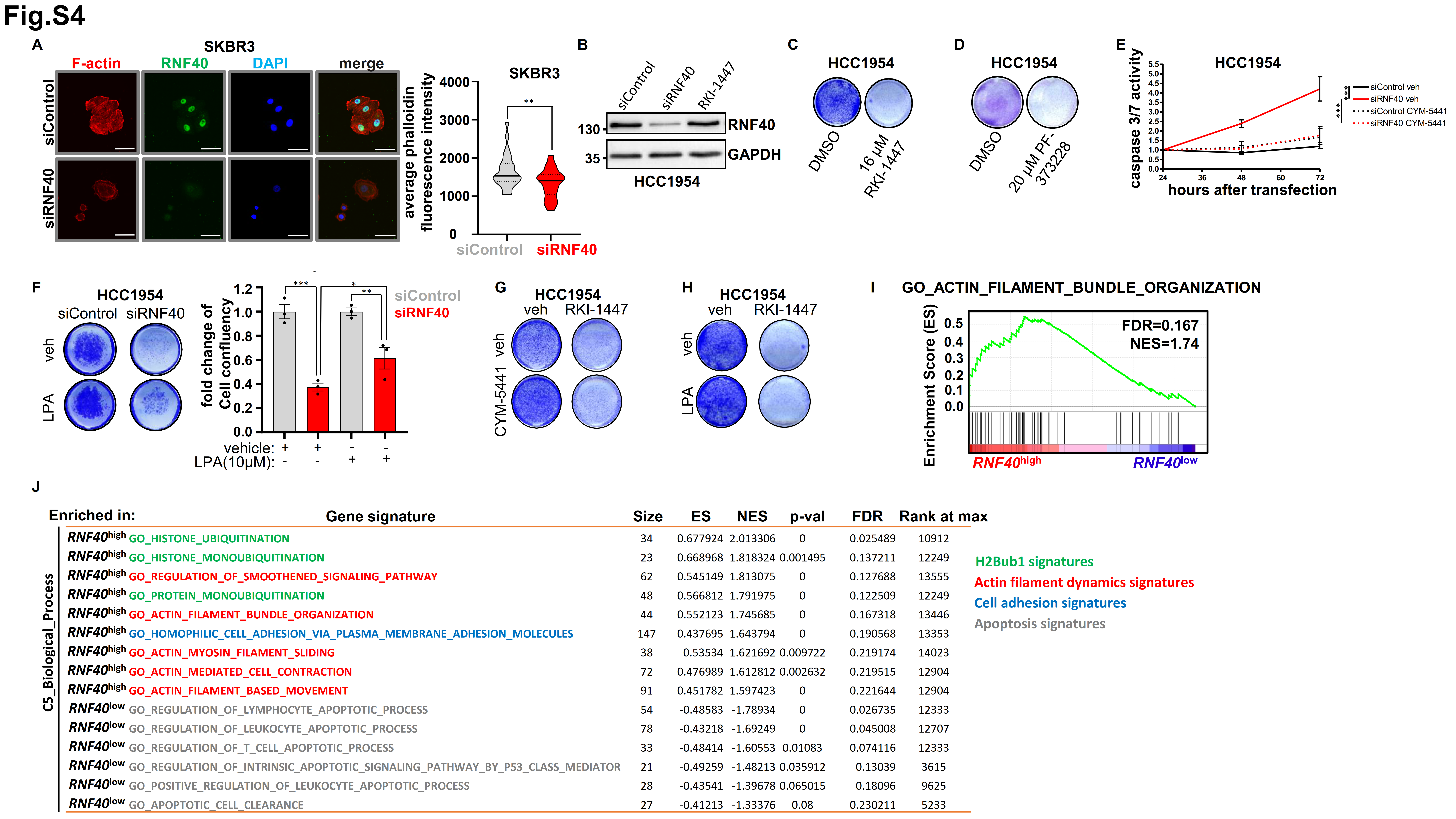

Supplement: Supplementary file 5 — Supplementary Figure S4 [file 41419_2020_3081_MOESM5_ESM.tif]

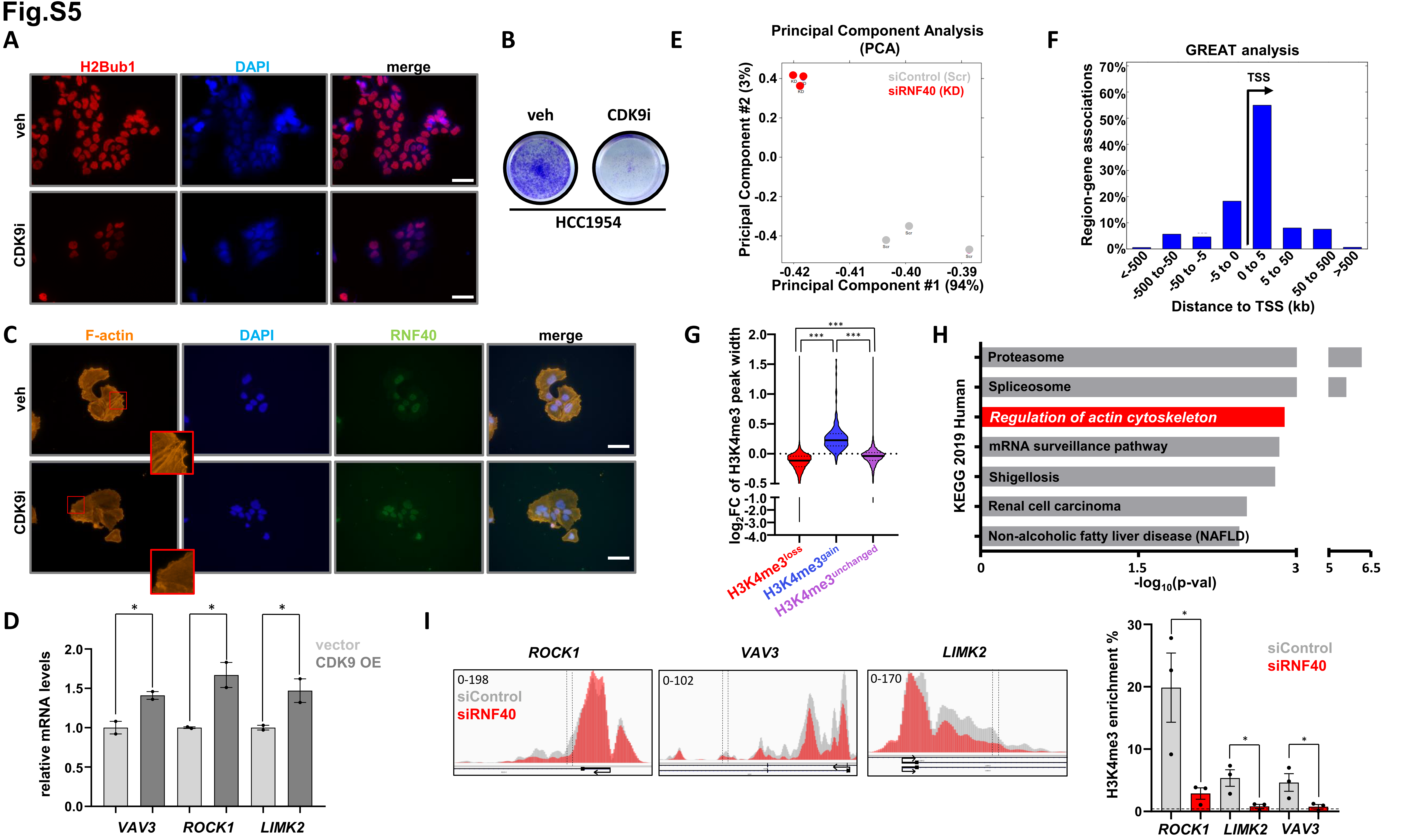

Supplement: Supplementary file 6 — Supplementary Figure S5 [file 41419_2020_3081_MOESM6_ESM.tif]

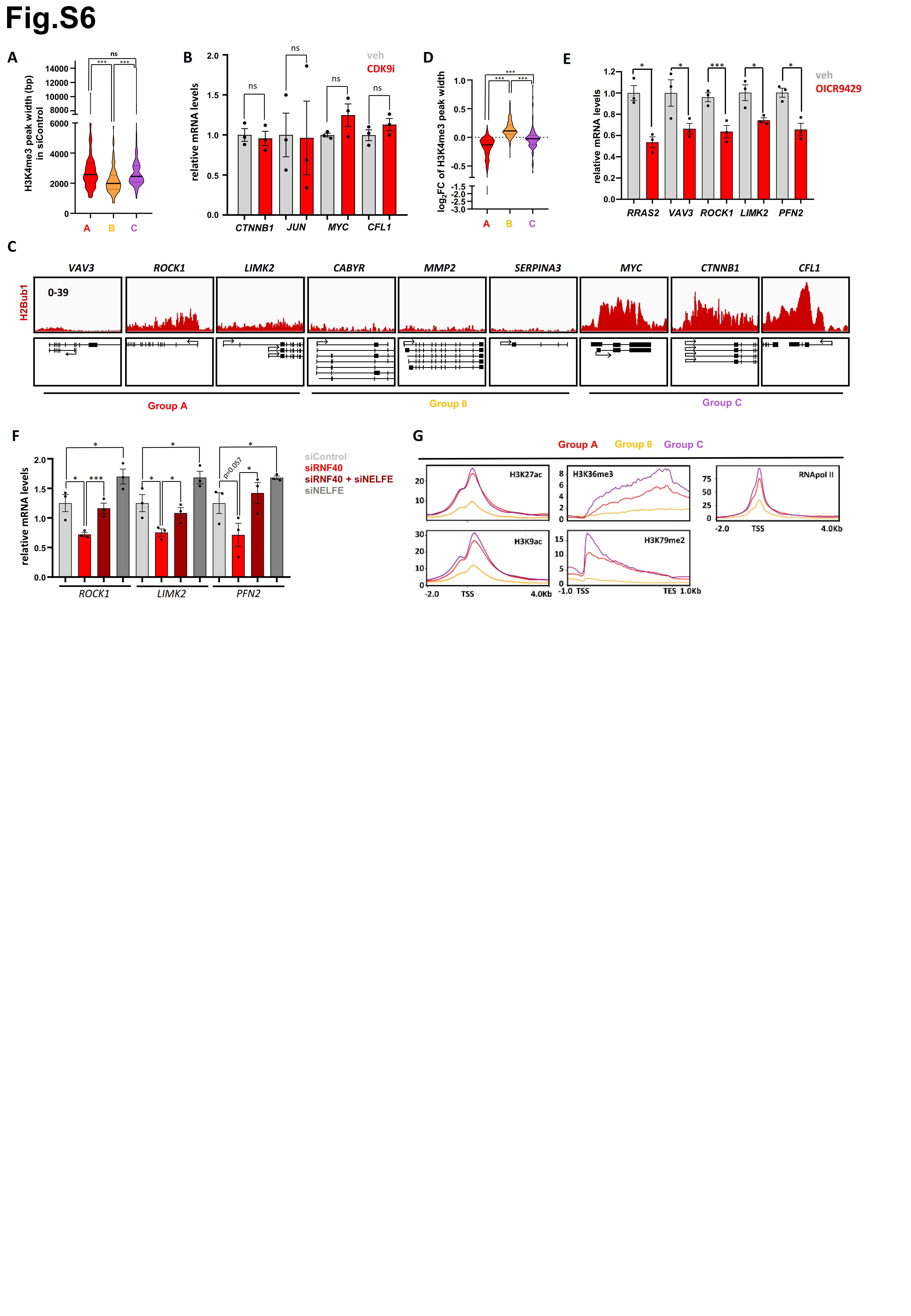

Supplement: Supplementary file 7 — Supplementary Figure S6 [file 41419_2020_3081_MOESM7_ESM.tif]
